# IL-27 disturbs lipid metabolism and restrains mitochondrial activity to inhibit γδ T17 cell-mediated skin inflammation

**DOI:** 10.1038/s41419-024-06887-0

**Published:** 2024-07-09

**Authors:** Mingyue Zhang, Dehai Li, Jing Zhu, Xue Xia, Hua Zhang, Jie Wu, Shengli Wang, Anyi Deng, Qiong Wen, Jingyi Tan, Jianlei Hao, Jun Jiang, Xiucong Bao, Guodong Sun, Jiajing Lu, Quanli Yang, Hengwen Yang, Guangchao Cao, Zhinan Yin, Qian Wang

**Affiliations:** 1 Guangdong Provincial Key Laboratory of Tumor Interventional Diagnosis and Treatment, Zhuhai Institute of Translational Medicine, Zhuhai People’s Hospital (Zhuhai Clinical Medical College of Jinan University), Jinan University, Zhuhai, 519000 China; 2 State Key Laboratory of Bioactive Molecules and Druggability Assessment, The Biomedical Translational Research Institute, Health Science Center (School of Medicine), Jinan University, Guangzhou, 510632 China; 3 Key Laboratory of Viral Pathogenesis & Infection Prevention and Control (Jinan University), Ministry of Education, Guangzhou, 510632 China; 4 Tianjian Laboratory of Advanced Biomedical Sciences, Institute of Advanced Biomedical Sciences, Zhengzhou University, Zhengzhou, 450001 China; 5 Department of Metabolic and Bariatric Surgery, The First Affiliated Hospital of Jinan University, Guangzhou, 510632 China; 6 School of Biomedical Sciences, Li Ka Shing Faculty of Medicine, The University of Hong Kong, Hong Kong SAR, China; 7 Guangdong Provincial Key Laboratory of Spine and Spinal Cord Reconstruction, The Fifth Affiliated Hospital (Heyuan Shenhe People’s Hospital), Jinan University, Heyuan, 517000 China; 8 Department of Dermatology, Shanghai Skin Disease Hospital, School of Medicine, Tongji University, Shanghai, 200443 China; 9 Institute of Psoriasis, School of Medicine, Tongji University, Shanghai, 200443 China

**Keywords:** Gammadelta T cells, Psoriasis, Interleukins

## Abstract

IL-17+ γδ T cells (γδ T17) are kick-starters of inflammation due to their strict immunosurveillance of xenobiotics or cellular damages and rapid response to pro-inflammatory stimulators. IL-27 is a well-recognized pleiotropic immune regulator with potent inhibitory effects on type 17 immune responses. However, its actions on γδ T17 mediated inflammation and the underlying mechanisms are less well understood. Here we find that IL-27 inhibits the production of IL-17 from γδ T cells. Mechanistically, IL-27 promotes lipolysis while inhibits lipogenesis, thus reduces the accumulation of lipids and subsequent membrane phospholipids, which leads to mitochondrial deactivation and ensuing reduction of IL-17. More importantly, *Il27ra* deficient γδ T cells are more pathogenic in an imiquimod-induced murine psoriasis model, while intracutaneous injection of rmIL-27 ameliorates psoriatic inflammation. In summary, this work uncovered the metabolic basis for the immune regulatory activity of IL-27 in restraining γδ T17 mediated inflammation, which provides novel insights into IL-27/IL-27Ra signaling, γδ T17 biology and the pathogenesis of psoriasis.

## Introduction

γδ T cells are T cells that bear γδ TCRs but have strong innate immune properties. These cells are rare in circulation but abundant in barrier tissues such as skin and intestine [1, 2]. Frequently encountering xenobiotic invasions in these tissues, γδ T cells are poised to surveil pathogens and cellular damages and have rapid reactions to these dangers. Normally, these first lines of immune responses mediated by γδ T cells are protective to eliminate invaders or damaged cells, however, they can be pathogenic and initiate proinflammatory reactions when facing excessive or persistent stimulations, which will further endanger tissues [3, 4]. γδ T17 (IL-17A+ γδ T) cells, for example, are located beneath the epithelial barrier, secret IL-17A when stimulated that induce chemokines and recruit neutrophils or adaptive immune cells to eliminate invasive pathogens. Excessive activation of γδ T17 cells will kick-start proinflammatory responses such as skin psoriatic inflammation [5].

It is conceivable that γδ T cells have vigorous anabolism and require a vast amount of energy to support their rapid reactions. We and other groups have demonstrated that γδ T cells express high levels of mTOR complex components and metabolic enzymes to meet the enormous demand for energy and mass [6–8]. It is now clear that γδ T17 cells have increased mitochondrial mass and activity and strongly engage oxidative metabolism [8–10]. Interfering mitochondria or mitochondrial oxidative reactions via targeting mTORC2, tricarboxylic acid cycle (TCA cycle), ATP et al., are valid strategies to restrict psoriasis progression in mouse models [6, 9–11].

IL-27 is an immune-regulatory cytokine with pleiotropic functions and has vigorous activities in barrier tissues [12]. It has potent inhibitory effects on Th17-mediated immune responses [13], but its actions on γδ T17 cells are less well understood. Besides, when dissecting the molecular basis of the inhibitory effects of IL-27, previous studies have focused on the induction of inhibitory factors such as IL-10 and PD-L1 or the antagonistic effects of STAT1 versus RORγt pathways [12], however, the metabolic basis involved received much less attention.

In this work, we find that γδ T cells express high levels of IL-27Ra and IL-27 inhibits γδ T17 cells mediated proinflammatory reactions. At the molecular level, IL-27 restrains lipogenesis in these cells and reduces phospholipids such as phosphatidylcholines, phosphatidylethanolamine and cardiolipin, which are essential components for organelle membrane and cytomembrane, especially for mitochondria. IL-27 reduces the mass and oxidative activity of mitochondria, and therefore inhibits the generation of γδ T17 cells and psoriatic inflammation.

## Materials and methods

### Reagents

Calcium-free RPMI 1640 medium (Cat no. GNM3187C) was purchased from GENOM. Fetal Bovine Serum for T cell (Cat no. SA101.02) was purchased from Cellmax. InVivoMab anti-mouse TCR γ/δ(Clone UC7, Cat no. BE0070), InVivoMab anti-mouse CD28 (Clone PV-1, Cat no. BE0015-5), InVivoPlus anti-mouse IFN-γ (Clone XMG1.2, Cat no. BP0055) and InVivoMab anti-mouse CD3ε (Clone 145-2C11, Cat no. BE0001-1 were purchased from BioXcell. Recombinant Mouse IL-27 (carrier-free, Cat no. 577408), Recombinant Mouse IL-23 (carrier-free, Cat no. 589006), Recombinant Mouse IL-1β (carrier-free, Cat no. 575106), Biotin anti-mouse TCR β chain Antibody (Cat no. 109204), Biotin anti-mouse TCR γ/δ Antibody (Cat no. 118103), Biotin anti-mouse CD19 Antibody (Cat no. 115504), Biotin anti-mouse/human CD11b Antibody (Cat no. 101204), Biotin anti-mouse CD11c Antibody (Cat no. 117304), Biotin anti-mouse CD8α Antibody (Cat no. 101204), Biotin anti-mouse CD4 Antibody (Cat no. 100704), APC-Cy7 anti-mouse CD45 (Cat no. 103116), PE-Cy7 anti-mouse CD3ε (Cat no. 100320), PerCP/Cy5.5 anti-mouse CD8α (Cat no. 100734), APC anti-mouse CD62L (Cat no. 104412), PE anti-mouse CD44 (Cat no. 103024), APC anti-mouse CD3ε (Cat no. 100312), PE-Cy7 anti-mouse IFN-γ (Cat no. 505826), PerCP/Cy5.5 anti-mouse IL-17A (Cat no. 506920), BV510 anti-mouse CD45 (Cat no. 103138) and APC-Cy7 Anti-mouse CD3ε (Cat no. 100222) were purchased from BioLegend. PE anti-mouse RORγt (Cat no. 12-6981-82), eFluro450 anti-mouse TCRγδ (Cat no. 48-5711-82) and FOXP3/TRN FACTOR STAIN BUFFER SET (Cat no. 00-5523-00) were purchased from eBioscience. FITC anti-mouse CD4 (Cat no. M10043-02E) was purchased from SungeneBiotech. Imiquimod (IMQ) cream (5%) was purchased from Sichuan Med-shine Pharmaceuticals. Seahorse XF Cell Mito Stress Test Kit (Cat no. 103015-100), Seahorse XFe96 FluxPak mini (Cat no. 102601-100), Seahorse XF 200 mM glutamine solution (Cat no. 103579-100), Seahorse XF DMEM (Cat no. 103575-100) and Seahorse XF 1.0 M glucose solution (Cat no. 103577-100) were purchased from Agilent. L-glutamine (Gln, Cat no. ST1441), Mito-Tracker Deep Red FM (C1032-250μg) and Mitochondrial membrane potential assay kit with JC-1 were purchased from Beyotime. LIVE/DEAD (Cat no. L34966) was purchased from Invitrogen. Cell Tak (Cat no. 354240) was purchased from Corning. Sodium pyruvate (Cat no. 11360070) and BODIPY™ 581/591 C11 (Cat no. D3821) were purchased from ThermoFisher. ATGL Antibody (Cat no. 2138), Phospho-DRP1 (Ser637) Antibody (Cat no. 4867), Phospho-DRP1 (Ser616) Antibody (Cat no. 3455), DRP1 Antibody (Cat no. 8570 S), Mitofusin-1 Antibody (Cat no. 14739), Mitofusin-2 Antibody (Cat no. 9482), OPA1 Rabbit mAb (Cat no. 80471), Tom20 Antibody (Cat no. 4904), SDHA Antibody (Cat no. 11998), UQCRFS1/RISP Antibody (Cat no. 95231), Cytochrome c Antibody (Cat no. 11940), COX1/MT-CO1 Antibody (Cat no. 55159), COX IV Antibody (Cat no. 4850), COX10 Antibody (Cat no. 24744), GAPDH Antibody (Cat no. 5174 S), Phospho-Stat3 (Ser727) Antibody (Cat no. 9134), Stat3 Antibody (Cat no. 4904), ATGL Antibody (Cat no. 2138), HSL Antibody (Cat no. 4107), Phospho-HSL (Ser563) Antibody (Cat no. 4139), Stat1 Antibody (Cat no. 9172), Phospho-Stat1 (Ser727) Antibody (Cat no. 8826), Phospho-Stat1 (Tyr701) Antibody (Cat no. 9167), Fatty Acid Synthase (FASN) Rabbit mAb (Cat no. 3180), p38 MAPK Antibody (Cat no. 8690 S), Phospho-p38 MAPK (Thr180/Tyr182) Antibody (Cat no. 9215 S) and BNIP3 Antibody (Cat no. 44060) were purchased from Cell Signaling Technology (CST). PINK1 Antibody (Cat no. BC100-494) was purchased from Novus Biologicals. FUNDC1 Antibody (Cat no. AP17377a) was purchased from Abcepta. TIM23 Antibody (Cat no. ab230253) was purchased from Abcam. HRP-mouse Anti-Rabbit IgG (L) Specific (Cat no. SA00001-7L) was purchased from Proteintech. Oligomycin A (MCH 32, Cat no. S1478) was purchased from Selleck. Ionomycin (Cat no. I9657), DNase I (Cat no. DN25-1G) Collagenase VIII (Cat no. C-2139), DMSO (Cat no. D2650), PMA (Cat no. P8139) were purchased from Sigma-Aldrich. Golgi-Plug (Cat no. 555029), Golgi stop (Cat no. 554724) and IMag™ Streptavidin Particles Plus – DM (Cat no. 557812) were purchased from BD. Red blood cell lysate (Cat no. 4992957) and Trizol Universal total RNA extraction reagent (Cat no. DP424) were purchased from Tiangen. Fludarabine (F-ara-A, NSC 118218, Cat no. HY-B0069) was purchased from MedChemExpress (MCE). C188-9 (Cat No. S8605) was purchased from Selleck. SDS-PAGE Protein loading buffer (5×) (Cat no. BL502A) was purchased from Biosharp. Reverse transcription Kit (Degenomics, Cat no. RR047B) and TB Green® Premix Ex Taq™ II (Tli RNaseH Plus, Cat no. RR820A) were purchased from Takara.

### Mice

B6.Cg-Tg(CD2-icre)4Kio/J (*Cd2-Cre*, Strain #:008520, RRID: IMSR_JAX:008520) and B6.129P2-*Tcrd*^*tm1Mom*^/J (*Tcrd-/-*, Strain #:002120, RRID: IMSR_JAX:002120) mice were purchased from the Jackson Laboratory and inbreed in our facility. *Il27ra*^*flox/flox*^ mice were generated in our lab [14] and wild-type C57BL/6 J mice (WT) were obtained from Beijing HFK Bioscience Company. All mice were hosted in specific pathogen-free conditions. Male mice weighing between 20–25 grams and aged 7–9 weeks were used for all animal experiments. Animal experiments were performed according to ethical regulations and protocols approved by the Institutional Animal Care and Use Committee of Jinan University.

### IMQ-induced psoriasis-like mouse model

The procedure was initiated with an intraperitoneal injection of tribromoethanol anesthesia (250–300 μl/mouse), followed by positioning them dorsal-side up on the experimental table. Subsequently, a region in the central upper back was shaved using an electric hair clipper, covering an area of 2 cm × 3 cm. Veet depilatory cream was then uniformly applied to the shaved area, left for 2 min, wiped off with a damp sterile cotton ball, and residual cream was thoroughly removed from the skin using multiple damp sterile cotton balls. Within 48 h post-depilation, a gentle and uniform application of IMQ cream was applied to the depilated area of the mice. Application occurred once daily at consistent time points, utilizing 62.5 milligrams of IMQ cream each time, continuously for 5–7 days. Before each application, mice were weighed, and their disease progression was evaluated.

The scoring criteria for psoriasis were as follows:

Erythema Severity: 0 = None, 1 = Slight, 2 = Moderate, 3 = Severe, 4 = Very Severe;

Skin Thickness: 0 = None, 1 = Slight, 2 = Moderate, 3 = Severe, 4 = Very Severe;

Scale Score: 0 = None, 1 = Slight, 2 = Moderate, 3 = Severe, 4 = Very Severe.

### Mouse splenic γδ T cells enrichment via magnetic-activated cell sorting (MACS)

Prepare R2F (1640 medium: FBS = 50:1, v/v). Isolate mouse spleen cells and discard supernatant post-centrifugation. Resuspend cells in red blood cell lysis buffer for 5–10 min, stop lysis with 5x R2F, centrifuge and discard the supernatant. Prepare antibody dilution: dilute Biotin-anti-mouse γδ antibody in 1640 medium (1:100). Resuspend cells in diluted antibody, adjust to 1.0 × 10^8^ cells/mL, incubate (4 °C, dark) for 30–45 min, centrifuge, discard supernatant, resuspend in 1640 medium. Use BD IMag™ Streptavidin Particles Plus - DM (vortex 1 min), adjust cells to 4.0 × 10^8^ cells/mL, and incubate (4 °C, dark, vortex every 10 min) for 30 min. Add 1640 medium, transfer to a flow tube, place on a sorting magnet (room temp, 10 min), remove supernatant, and resuspend the pellet in the medium in a new tube. Adjust with R2F, centrifuge, discard supernatant. Resuspend in R2F, centrifuge, discard supernatant. The pellet represents enriched γδ T cells.

### Mouse splenic naïve γδ T cells isolation

Prepare R2F: 1640 medium to FBS ratio of 50:1. Spleens were dissociated into single-cell suspensions, and supernatants were removed post-centrifugation. Cells were resuspended in red blood cell lysis buffer, lysed for 5–10 min, and the lysis was halted by adding 5 times R2F. Supernatants were discarded post-centrifugation. Prepare antibody dilution: 1640 medium: Biotin-conjugated antibody cocktail (anti-mouse CD4, CD19, CD8, CD11b and CD11c) = 100:1. Resuspend cells in the antibody dilution, adjust to 1.0 × 10^8^ cells/mL, incubate at 4 °C in the dark for 30–45 min, and remove supernatants post-centrifugation. Resuspend cells in 1640 medium and remove supernatants after centrifugation. Resuspend cells in BD IMag™ Streptavidin Particles Plus - DM magnetic bead solution, adjust to 4.0 × 10^8^ cells/mL, incubate at 4 °C in the dark for 30 min (shaking every 10 min), add 5 times 1640 medium, place on a sorting magnet for 10 min, aspirate and transfer supernatants, replenish with R2F, and discard supernatants post-centrifugation. Resuspend cells in R2F, and discard supernatants after centrifugation. Prepare flow cytometry antibody dilution: 1×PBS: fluorochrome-conjugated antibody cocktail (anti-mouse CD3ε, CD44, CD62L and γδ TCR) = 100:1. Resuspend cells in the prepared antibody dilution, store in a dark, chilled container for 15–20 min. After washing by 1xPBS, naïve γδ T cells (CD3ε^+^ TCRδ^+^ CD44^low^ CD62L^high^ subset) were sorted using FACSAria cell sorter.

### In vitro differentiation of mouse γδ T17 cells

Purified anti-mouse γδ TCR (Clone: UC7-13D5, 10 μg/ml) was precoated onto a 48-well plate. Mouse splenic cells (2.5 × 10^6^ cells/mL) were cultured in RPMI 1640 medium containing 10% FBS and supplemented with anti-mouse CD28 (Clone: PV-1, 1 μg/mL), rmIL-1β (10 ng/mL), rmIL-23 (10 ng/mL) and anti-IFN-γ blocking Abs (Clone: XMG1.2, 10 μg/mL). Cell cultures were maintained in the 48-well plate with a total volume of 0.5 ml per well. Following fresh media replacement on the third day, the cells were further differentiated until the fifth to the sixth day. Total γδ T cells after differentiation were enriched via MACS as mentioned above. Naïve γδ T cells (1 × 10^6^ cells/mL) were also used for in vitro γδ T17 differentiation by a similar protocol without MACS enrichment. IL-27 (50 ng/ml), Forskolin (10 μM), Oligomycin A (10 μM), STAT1 inhibitor (Fludarabine, 10 μM) or STAT3 inhibitor (C188-9, 10 μM) was added in the first 3 days of culture for some experiments.

### Flow cytometry analysis

Single-cell suspensions were used for staining antibodies and analyzed using the BD FACS Verse Flow Cytometer (BD), the FACS data were analyzed with FlowJov.10 software.

### RNA sequencing

Transcriptomic sequencing and analysis were conducted by OE Biotech Co., Ltd. (Shanghai, China). RNA Extraction: Total cellular RNA was extracted using the Trizol method. RNA Purity and Quantification: The NanoDrop 2000 spectrophotometer was used for RNA assessment and quantification. RNA Integrity: RNA integrity was evaluated using the Agilent 2100 Bioanalyzer. Library Construction: Transcriptional libraries were constructed using the VAHTS Universal V6 RNA-seq Library Prep kit following the manufacturer’s instructions. Sequencing: Libraries were sequenced using the Illumina Novaseq 6000 platform, generating 150 bp paired-end reads. Raw reads in fastq format were processed using FASTQC software to remove low-quality reads, obtaining clean reads for subsequent analysis. HISAT2 software was utilized for reference genome alignment, and gene expression levels (FPKM) were calculated. HTSeq-count was used to obtain read counts for each gene. Gene counts underwent PCA analysis and visualization using R (v 3.2.0) to assess biological replicates. DESeq2 software was employed for differential gene expression analysis. Genes meeting *q* value < 0.05 and fold change > 2 or < 0.5 were defined as Differentially Expressed Genes (DEGs). DEGs underwent enrichment analysis using the hypergeometric distribution algorithm for GO, and KEGG Pathways. GSEA software was applied for gene set enrichment analysis. RNA seq data were deposited at the NCBI database to be publicly available with accession numbers SRR27385675-SRR27385678 and SRR27385153-SRR27385157.

### Lipidomic analysis using LC-MS

Shanghai Luming Biological Technology Co., Ltd. (Shanghai, China) conducted the metabolomic data analysis. Initial Q Exactive LC-MS data in raw format underwent processing using LipidSearch software to derive MSn and precise mass-to-charge ratios (m/z) of parent ions. Identification of lipid molecular structures and their positive and negative ion adduct patterns relied on individual sample parent ions and multi-stage mass spectrometry data. Results were arranged within specified retention time windows and consolidated into a unified report, forming the primary data matrix. Normalization of all peak signals within each sample involved converting signal intensities to relative values within the spectrum, subsequently scaled by a factor of 10,000. Subsequent data processing entailed eliminating peaks with missing values (ion intensity = 0) exceeding 50% in sample groups, replacing zero values with half the minimum detected value. Merging positive and negative ion data generated a comprehensive data matrix imported into R for Principal Component Analysis (PCA), enabling an overview of sample distributions and analysis stability. Orthogonal Partial Least Squares Discriminant Analysis (OPLS-DA) and Partial Least Squares Discriminant Analysis (PLS-DA) facilitated the identification of differing metabolites between groups. To mitigate overfitting, 7-fold cross-validation and 200 Response Permutation Tests (RPT) assessed model quality. Variable Importance in Projection (VIP) values, derived from the OPLS-DA model, ranked each variable’s overall contribution to group discrimination. Subsequently, a two-tailed Student’s *t*-test confirmed the significance of inter-group differences in metabolites. Lipidomic data were deposited at MetaboLights database to be publicly available with accession numbers MTBLS9272.

### Western blot analysis

Cell samples were rinsed twice with pre-chilled 1× PBS, followed by centrifugation to remove the supernatant. Then, the samples are resuspended in an appropriate volume of RIPA lysis buffer and left to lyse on ice for 30–60 min with intermittent vortexing. After centrifugation, the supernatant is collected for protein concentration determination using a BCA assay. The remaining sample is mixed with 5× loading buffer, heated at 95 °C for 10 min, and stored at −80 °C. For the Western Blot, markers and samples are loaded sequentially. Gel electrophoresis runs at 60–80 V initially and switches to 100 V post-separation. Proteins are transferred onto a PVDF membrane, which is then blocked with 5% skimmed milk in 1× TBST for 60 min. Desired sections are incubated in primary antibody dilution overnight at 4 °C, followed by washes and incubation with a secondary antibody. Chemiluminescent substrate (A: B ratio 1:1) is used for protein detection, with imaging via a gel documentation system. If needed, the PVDF membrane would be incubated in a strip solution to remove exited antibodies before assessing a different protein with adjacent molecular weight. The full and uncropped western blots were available in the Supplemental Material.

### Histology

The skin tissue is initially fixed in 4% paraformaldehyde at room temperature. Subsequently, it undergoes dehydration, embedding in paraffin wax, and sectioning. Post-sectioning, the paraffin-embedded tissue sections are deparaffinized, followed by staining with hematoxylin and eosin. Following staining, dehydration and mounting occur, followed by microscopic examination and image acquisition for analysis, where the cell nuclei appear blue and the cytoplasm appears red.

### Transmission electron microscopy of mitochondria

#### Tissue fixation

Centrifuge suspended cells, discard supernatant, resuspend cells in electron microscopy fixative, fix at room temperature in darkness for 2 h, then transfer to 4 °C in darkness for storage.

#### Agar pre-embedding

Discard supernatant after centrifugation, resuspend cells in 0.1 M phosphate buffer (pH 7.4), rinse for 3 min at room temperature in darkness (repeat 3 times), prepare 1% agar solution, cool, add to Eppendorf tubes, swiftly suspend the pellet in agar.

#### Fixation

Embed agar blocks in 1% osmium tetroxide at room temperature in darkness for 2 h.

#### Washing

Rinse thrice with 0.1 M phosphate buffer (pH 7.4) for 15 min each time.

#### Dehydration

Gradually place samples in 30–50–70–80–95–100–100% ethanol for 20 min each, followed by two changes of 100% acetone for 15 min each.

#### Embedding

Use 1:1 acetone:812 embedding medium for 3 h at 37 °C; 1:2 acetone:812 embedding medium at 37 °C overnight; pure 812 embedding medium at 37 °C for 6 h; pour pure 812 embedding medium into embedding molds, insert samples, and incubate in an oven at 37 °C overnight.

#### Polymerization

Polymerize embedding molds in an oven at 60 °C for 48 h and retrieve resin blocks.

#### Ultra-thin sectioning

Cut 60–80 nm ultra-thin sections using a diamond knife on 150-mesh copper grids.

#### Staining

Stain copper grids in 2% uranyl acetate saturated alcohol for 8 min in darkness; rinse thrice in 70% ethanol, thrice in ultrapure water; stain in 2.6% lead citrate solution avoiding carbon dioxide for 8 min; rinse thrice in ultrapure water, blot dry, place copper grids in grid boxes at room temperature overnight. Observation with Transmission Electron Microscopy, image acquisition, and analysis.

### Statistics and reproducibility

All experiments and data in this study were repeated independently at least twice, consistently yielding coherent results. The presented data were acquired from biologically independent samples. GraphPad Prism (v.8) was used for both graphical representation and statistical analysis. Detailed descriptions of the statistical tests conducted were provided in the figure legends, conforming to assumptions of similar variances and normal distribution. Sample sizes were not predetermined using specific statistical methods. No specific method of randomization was used to determine how samples/animals were allocated to experimental groups and processed. No sample was excluded from the analysis. The investigators were not blinded to the group allocation during the experiment and/or when assessing the outcome. Two-group comparisons were conducted using a two-tailed unpaired Student’s *t*-test. Assessments involving more than two groups utilized one-way analysis of variance (ANOVA). Two-way ANOVA was used to analyze interactions between two independent variables, complemented by Sidak’s multiple comparison test for specific time point differences. Data are presented as mean ± standard error of the mean (s.e.m.) unless otherwise specified. Statistical significance was considered at *P* < 0.05.

## Results

### IL-27 signaling inhibits the generation of γδ T17 cells

**Fig. 1:**
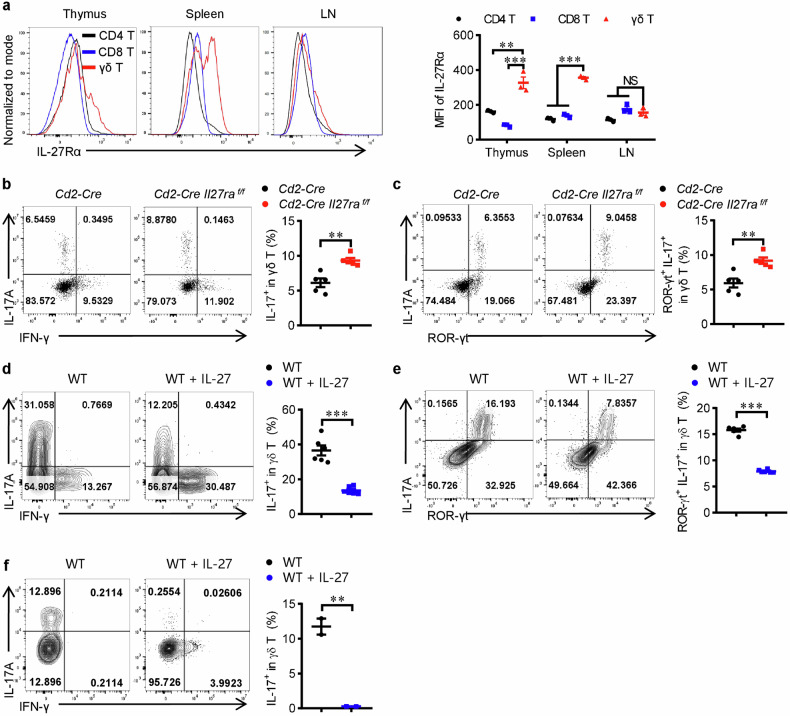
IL-27 signaling inhibits the generation of γδ T17 cells. **a** The expression of IL-27Ra in CD4^+^, CD8^+^ and γδ T cells from the thymus, spleen or lymph nodes of C57BL/6 J mice was detected by flow cytometry (*n* = 3). **b** Flow cytometry analysis of IL-17A production by spleen γδ T cells from *Cd2-Cre* and *Cd2-Cre Il27ra*^*flox/flox*^ mice (*n* = 5). **c** Flow cytometry analyses of RORγt^+^ IL-17A^+^ proportion in spleen γδ T cells from *Cd2-Cre* and *Cd2-Cre Il27ra*^*flox/flox*^ mice (*n* = 5). **d**, **e** Splenocytes from WT mice were cultured under γδ T17 priming conditions with or without rmIL-27 (50 ng/ml) for 3 days and then expended till Day 5 (**e**) or Day 6 (**d**), the production of IL-17A (**d**), or RORγt^+^ IL-17A^+^ percentages (**e**) in γδ T cells were detected and shown (*n* = 6). **f** Naive γδ T cells (CD44^low^ CD62L^high^) were sorted from spleens and peripheral lymph nodes of WT mice and polarized toward the γδ T17 condition with or without rmIL-27 (50 ng/ml). Flow cytometry analysis of IL-17 production (*n* = 3). Data were presented as mean ± SEM, statistical differences were performed using Two-tailed unpaired student’s *t*-test (**b**)–(**f**) or one-way ANOVA (**a**). **p < 0.01, ***p < 0.001, NS, not significant.

IL-27 signaling has potent inhibitory effects on Th17-mediated immune responses, but its role in γδ T17 cells is less well understood. To explore its actions on γδ T cells, we first detected the expression level of IL-27Ra, an essential and specific subunit of IL-27 receptor, in γδ T cells. Compared with CD4^+^ and CD8^+^ αβ T cells, mouse thymic and splenic γδ T cells express higher levels of IL-27Ra (Fig. 1a). Interestingly, the expression of IL-27Ra in γδ T cells from peripheral lymph nodes was relatively low compared with γδ T cells from the thymus and spleen and was comparable with αβ T cells (Fig. 1a). Usually, γδ T cells from peripheral lymph nodes expressed higher levels of IL-17A compared with those from spleen cells under homeostatic state [15] (Fig. 1b and Supplementary Fig. 1h). The low level of IL-27Ra in γδ T cells from peripheral lymph nodes suggests a less potential impact of IL-27 on these cells. We next sought to investigate the function of this signaling pathway in γδ T cells using a genetically deficient model, i.e. *Cd2-Cre Il27ra*^*flox/flox*^ mouse strain. The percentages of CD4^+^, CD8^+^ αβ T cells and γδ T cells in the thymus of *Cd2-Cre Il27ra*^*flox/flox*^ mice were comparable with *Cd2-Cre* controls, the proportion of these cells in the peripheral lymph organs were largely unaffected either except for a slight increase of γδ T cells in the spleen (Supplementary Fig. 1a–f), suggesting that IL-27Ra signaling deficiency didn’t stunt the development or peripheral distribution of T cells compartments. Currently, two functional distinct γδ T cell subsets have been identified, i.e. IFN-γ + γδ T cells with cytotoxicity against tumor or intracellular infections and IL-17+ γδ T cells with proinflammatory or tissue repairing activity [2, 16]. Since IL-27 have potent regulatory effects on both IFN-γ+ and IL-17+ αβ T cells [12, 13], we then tested if Il27ra deficiency affects these two γδ T cell subsets. When analyzing cytokine production, the levels of IL-17A or IFN-γ in γδ T cells from adult thymus or peripheral lymph nodes were unaltered after *Il27ra* depletion, the proportion of IFN-γ^+^ γδ T cells in the spleen was unchanged either, while splenic γδ T17 cells were significantly elevated (Fig. 1b, c and Supplementary Fig. 1g–i), suggesting that IL-27Ra signaling specifically disfavors the generation of γδ T17 in the spleen. It’s worth noting that the nature occurring γδ T17 in peripheral tissues (including spleen) and circulation under homeostasis were developed in embryonic thymus [17], the above results didn’t rule out the possibility that IL-27 might regulate the embryonic development and peripheral distribution of nature-occurring γδ T17 cells. We then tested whether IL-27 inhibited the differentiation of splenic γδ T17 cells in vitro. Indeed, the addition of IL-27 protein significantly inhibited the priming of total or naïve WT splenic γδ T cells towards γδ T17 in vitro (Fig. 1d–f). IL-27 treatment also upregulated the percentages of γδ T1 cells in vitro (Supplementary Fig. 1j), suggesting that IL-27 might promote the priming of γδ T1 cells. The findings made in vitro were bona fide effects of IL-27 since treating *Il27ra* deficient splenic γδ T cells by IL-27 yielded no such alterations (Supplementary Fig. 1k). Collectively, a firm conclusion made from the above results was that IL-27 signaling inhibited the generation of splenic γδ T17 cells.

### IL-27 disturbs lipid metabolism and restrains the generation of phospholipids in γδ T17 cells

**Fig. 2:**
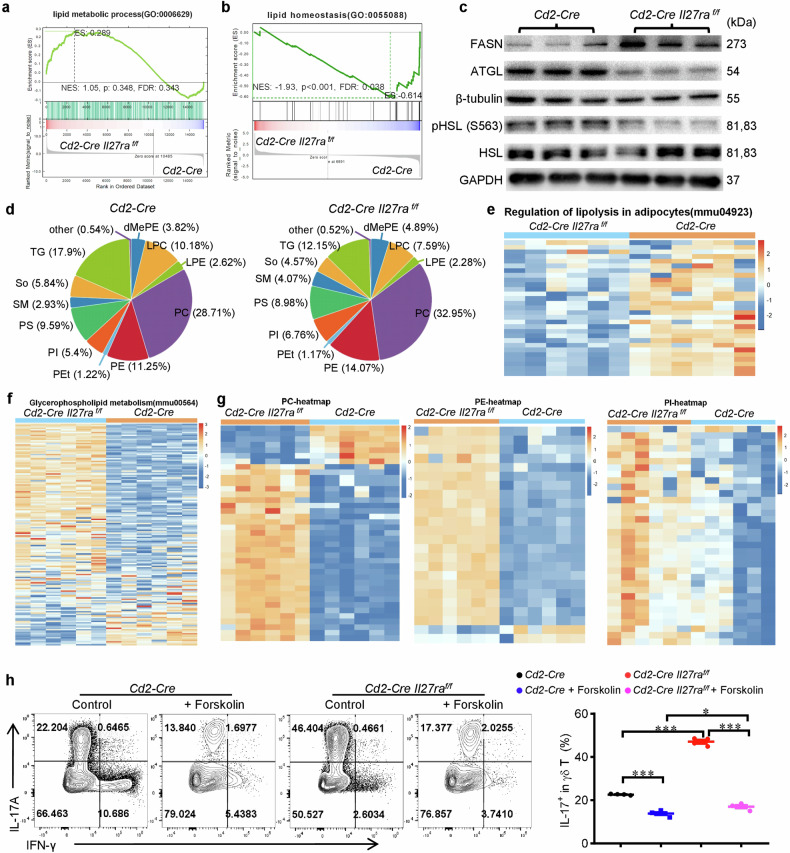
IL-27 disturbs lipid metabolism and restrains the generation of phospholipids in γδ T17 cells. **a**, **b** RNA-sequencing analysis was performed using in vitro differentiated γδ T17 cells from the spleen of *Cd2-Cre Il27ra*^*flox/flox*^ or *Cd2-Cre* mice. GSEA analysis of genes in lipid metabolic process (**a**) and lipid homeostasis (**b**) were shown. **c** Immunoblotting of in vitro differentiated γδ T17 cells from the spleen of *Cd2-Cre Il27ra*^*flox/flox*^ or *Cd2-Cre* mice (*n* = 3). **d**–**g** Lipids were extracted from in vitro differentiated γδ T17 cells from the spleen of *Cd2-Cre Il27ra*^*flox/flox*^ or *Cd2-Cre* mice and used for lipidomic analysis via LC-MS (*n* = 6). **d** Pie chart showing the proportions of each type of lipid component. TG triglyceride, So sphingosine, SM Sphingomyelin, PS Phosphatidylserine, PI phosphatidylinositol, PEt Phosphatidylethanol, PE phosphatidylethanolamines, PC phosphatidylcholine, LPE Lyso phosphatidyl ethanolami, LPC Lyso phosphatidylcholine, dMePE Dimethylphosphatidyleth anolamine. **e**, **f** Metabolite set enrichment analysis of significantly altered lipids involved in lipolysis (**e**) and glycerophospholipids (**f**). **g** Heatmap of significantly altered phospholipids belonging to PC, PE and PI. **h** Flow cytometry analysis of IL-17 in γδ T cells from *Cd2-Cre* and *Cd2-Cre Il27ra*^*flox/flox*^ mice that were cultured under γδ T17 priming condition with or without Forskolin (10 μM) (*n* = 4). Data were presented as mean ± SEM, statistical differences were performed using One-way ANOVA (**h**). **p < 0.01, ***p < 0.001.

To dissect the molecular mechanism underlying the inhibition of γδ T17 by IL-27, we performed RNA-sequencing analysis of in vitro differentiated γδ T17 cells that were isolated from the spleen of *Cd2-Cre Il27ra*^*flox/flox*^ or *Cd2-Cre* mice, and in vitro differentiated wild-type (WT) γδ T17 cells in the presence or absence of IL-27. Genetic deficiency of *Il27ra* slightly disturbed the transcriptome of γδ T cells, with 113 genes increased and 56 genes decreased (Supplementary Fig. 2a and Supplementary Table 1). Still, RNA-seq analysis confirmed the preference for γδ T17 cells after *Il27ra* deficiency (Supplementary Fig. 2b). IL-27 treatment in vitro had a much more profound impact on the transcriptome of γδ T17 cells, affecting 3217 genes with 2530 increased and 687 decreased (Supplementary Fig. 2c and Supplementary Table 2). Enrichment analysis of the differentially expressed genes revealed that IL-27 disturbs many metabolic processes, especially lipid metabolism and mitochondria-related oxidative metabolism (Fig. 2a, b and Supplementary Fig. 2d). These results were in line with previous reports that γδ T17 cells favor lipids and strongly engage oxidative metabolism [8]. Therefore, we next investigated whether IL-27 limits the generation of γδ T17 cells through these metabolic processes.

We first verified the effects of IL-27 on lipid metabolism. In vitro generated *Il27ra* deficient γδ T17 cells expressed a higher level of fatty acid synthase (FASN) but a much lower level of adipose triglyceride lipase (ATGL), the activation of hormone-sensitive lipase (HSL) was also significantly decreased in *Il27ra* deficient γδ T17 cells (Fig. 2c). These results suggested that IL-27 promotes lipolysis but inhibits de novo lipogenesis in γδ T17 cells. Then we performed lipidomic analysis of these in vitro generated *Il27ra* deficient γδ T17 cells and controls (Fig. 2d and Supplementary Table 3). Enrichment analysis of the altered lipids confirmed the reduction of lipolysis and augmentation of fatty acids such as polyunsaturated fatty acids after *Il27ra* depletion (Fig. 2e and Supplementary Fig. 3a–e). Interestingly, *Il27ra* deficient γδ T17 cells converted these augmented fatty acids into phospholipids, the main components of organelle membrane and cytomembrane, rather than energy-storing triglyceride (Fig. 2d, f, g and Supplementary Fig. 3f–i), which were in line with the immune rather than the energy-storing activity of γδ T cells. Therefore, we next test whether the altered lipid metabolism contributed to the inhibition of IL-17 by IL-27. We found that adding forskolin, an activator of lipolysis, significantly inhibited the in vitro differentiation of γδ T17 cells and reduced the differences between *Il27ra* deficient and sufficient γδ T17 cells (Fig. 2h). Collectively, these results indicated that the inhibition of γδ T17 by IL-27 was partially attributable to the disturbance of lipid metabolism.

### IL-27 downregulates mitochondrial mass and oxidative phosphorylation in γδ T17 cells

**Fig. 3:**
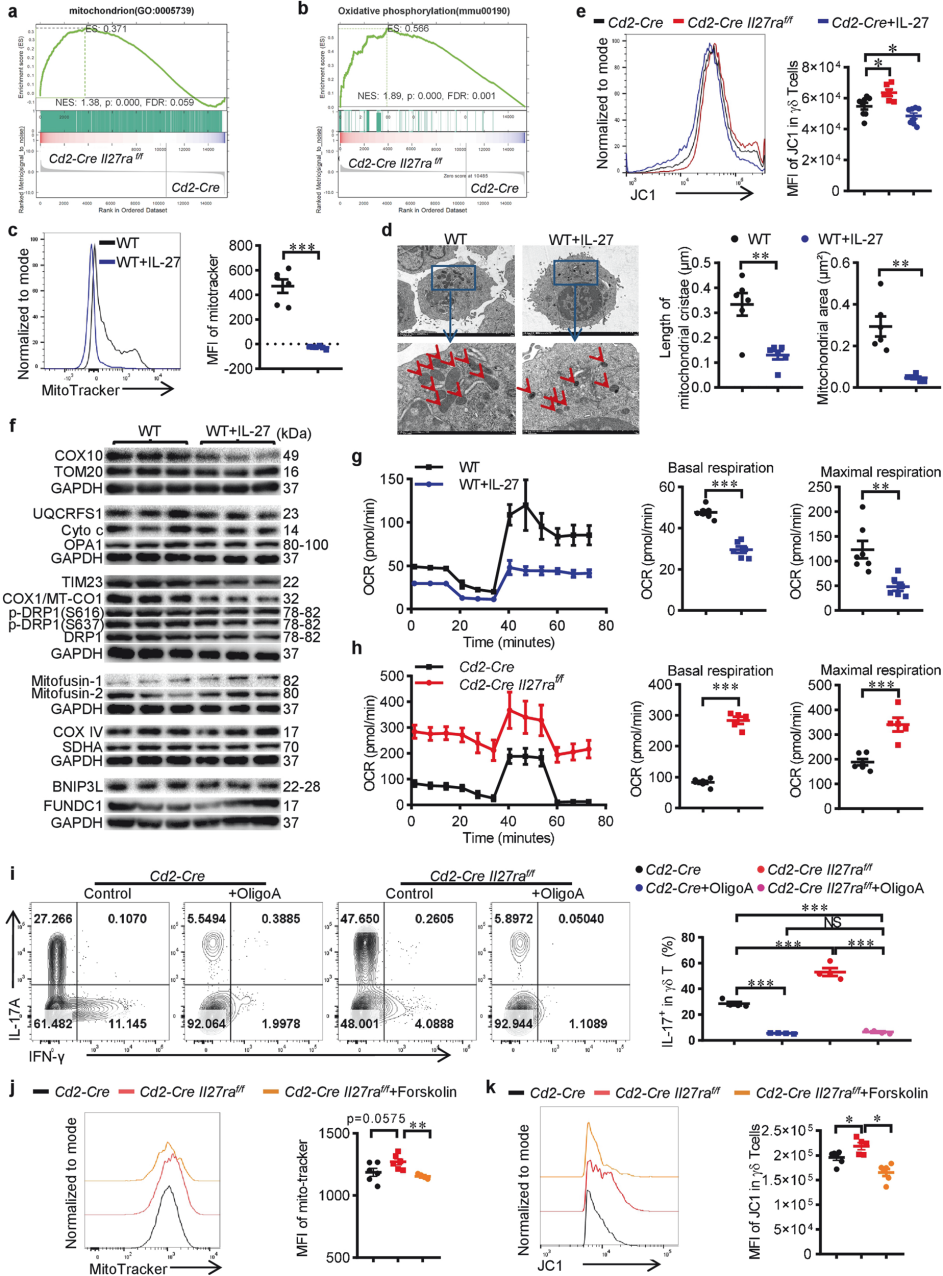
IL-27 reduces mitochondrial mass and impedes oxidative phosphorylation in γδ T17 cells. RNA-sequencing analysis was performed using in vitro differentiated γδ T17 cells from the spleen of *Cd2-Cre Il27ra*^*flox/flox*^ or *Cd2-Cre* mice. GSEA analysis of genes in mitochondrion (**a**) and oxidative phosphorylation (**b**) were shown. **c** MitoTracker staining of WT γδ T cells that were differentiated in vitro under γδ T17 condition with or without rmIL-27 (50 ng/ml) (*n* = 6). **d** Transmission electron microscopy of WT γδ T cells that were differentiated in vitro under γδ T17 condition with or without rmIL-27 (50 ng/ml). The size and morphology of mitochondria were determined and shown (*n* = 6). **e** JC-1 staining of γδ T cells from *Cd2-Cre Il27ra*^*flox/flox*^ (*n* = 6) or *Cd2-Cre* mice (*n* = 8) that were differentiated in vitro under γδ T17 condition with or without rmIL-27 (50 ng/ml). **f** Immunoblotting of mitochondrial proteins in WT γδ T cells that were differentiated in vitro under γδ T17 condition with or without rmIL-27 (50 ng/ml) (*n* = 3). **g** Oxygen consumption rate (OCR) of WT γδ T cells that were differentiated in vitro under γδ T17 condition with or without rmIL-27 (50 ng/ml) was determined via the Seahorse assay. Cumulative data for the basal and maximal respiration were shown. **h** Oxygen consumption rate (OCR) of in vitro differentiated γδ T17 cells from *Cd2-Cre Il27ra*^*flox/flox*^ (*n* = 5) or *Cd2-Cre* (*n* = 6) mice was determined via the Seahorse assay. Cumulative data for the basal and maximal respiration were shown. **i** Flow cytometry of IL-17 levels in γδ T cells from *Cd2-Cre Il27ra*^*flox/flox*^ or *Cd2-Cre* mice that were differentiated in vitro under γδ T17 condition in the presence or absence of Oligomycin A (10 μM) (*n* = 4). **j** MitoTracker staining of γδ T cells from *Cd2-Cre* (*n* = 6) or *Cd2-Cre Il27ra*^*flox/flox*^ mice that were differentiated in vitro with (*n* = 4) or without (*n* = 6) Forskolin (10 μM). **k** JC-1 staining of γδ T cells from *Cd2-Cre* (*n* = 6) or *Cd2-Cre Il27ra*^*flox/flox*^ mice that were differentiated in vitro with (*n* = 6) or without (*n* = 5) Forskolin (10 μM). Data were presented as mean ± SEM, statistical differences were performed using Two-tailed unpaired student’s *t*-test (**c**, **d**, **g**, **h**) or One-way ANOVA (**e**, **i**–**k**). *p < 0.05, **p < 0.01, ***p < 0.001, NS not significant.

As mentioned above, transcriptomic analysis suggested that IL-27 treatment inhibited oxidative phosphorylation (OXPHOS) in γδ T17 cells (Supplementary Fig. 2d). GSEA analysis of mitochondria and oxidative phosphorylation pathways in *Il27ra* deficient γδ T cells also supported the same conclusion (Fig. 3a, b). We then checked whether IL-27 / IL-27Ra signaling genuinely affects the mitochondria and oxidative metabolism. Indeed, IL-27 treated γδ T17 cells showed reduced mitochondrial mass, shortened cristae and shrunken size (Fig. 3c, d). The mitochondrial membrane potential was also slightly increased in *Il27ra* deficient γδ T17 cells but decreased in IL-27-treated WT cells (Fig. 3e). When analyzing the levels of mitochondrial proteins, we found that IL-27 treatment significantly reduced the expression of TIM23 and cytochrome oxidase COX10 and MT-Co1, while other proteins were largely unchanged or only marginally affected (Fig. 3f). TIM23 is a key component of the mitochondrial preprotein translocase that is located in the inner membrane and is essential for the import of mitochondrial preproteins and the biosynthesis of mitochondria [18]. The reduction of TIM23 might explain the reduced mitochondria mass after IL-27 treatment. COX10 and MT-Co1 are key components of cytochrome oxidase that are critical in the mitochondrial electron transport chain and oxidative phosphorylation reactions [19, 20], the downregulation of these proteins was consistent with the transcriptomic data and strongly suggested the inhibition of mitochondrial oxidative metabolism by IL-27. Indeed, the Seahorse Assay confirmed that IL-27 treatment inhibited the oxygen consumption rate (OCR) of γδ T17 cells while *Il27ra* deficiency potentiated it (Fig. 3g, h). To test whether the upregulation of mitochondrial activity after *Il27ra* depletion contributes to the elevation of IL-17 production, we added oligomycin A, a classical mitochondrial reagent that blocks mitochondrial ATP synthesis and oxidative metabolism [21], during in vitro differentiation of γδ T17 cells. As expected, oligomycin A significantly inhibited the differentiation of γδ T17 cells and eliminated the difference between *Il27ra* deficient and sufficient γδ T17 cells (Fig. 3i). Taken together, these findings converged to strongly support the conclusion that IL-27 reduces mitochondrial mass and impedes oxidative phosphorylation to inhibit γδ T17 cells.

Mitochondria are organelles with double layers of membrane and phospholipids are also the main lipid components of mitochondria membranes. These phospholipids are closely related to the regulation of mitochondrial membrane fluidity and interact with many enzymes and proteins in the mitochondria, thereby affecting mitochondrial function [22]. Besides, the inner membrane of mitochondria contains a specific phospholipid, i.e. cardiolipin, which plays a crucial role in maintaining the integrity and order of the mitochondrial inner membrane [23]. Cardiolipin is also an indispensable part of the energy production and oxidative phosphorylation process of mitochondria [24]. Since IL-27 disturbs lipid metabolism and restrains the generation of phospholipids including cardiolipin (Supplementary Fig. 3i), reduces mitochondrial mass and impedes oxidative metabolism, we next tested whether the disturbance of lipid metabolism contributed to the functional alteration of mitochondria. We found that inducing lipolysis by forskolin reversed the elevation of mitochondrial mass and mitochondrial membrane potential in *Il27ra* deficient γδ T17 cells (Fig. 3j, k), which was consistent with our hypothesis and suggested that the disturbance of lipid metabolism by IL-27 contributed to the functional alteration of mitochondria.

### IL-27 restrains lipid anabolism and mitochondrial activity partially via STAT1

**Fig. 4:**
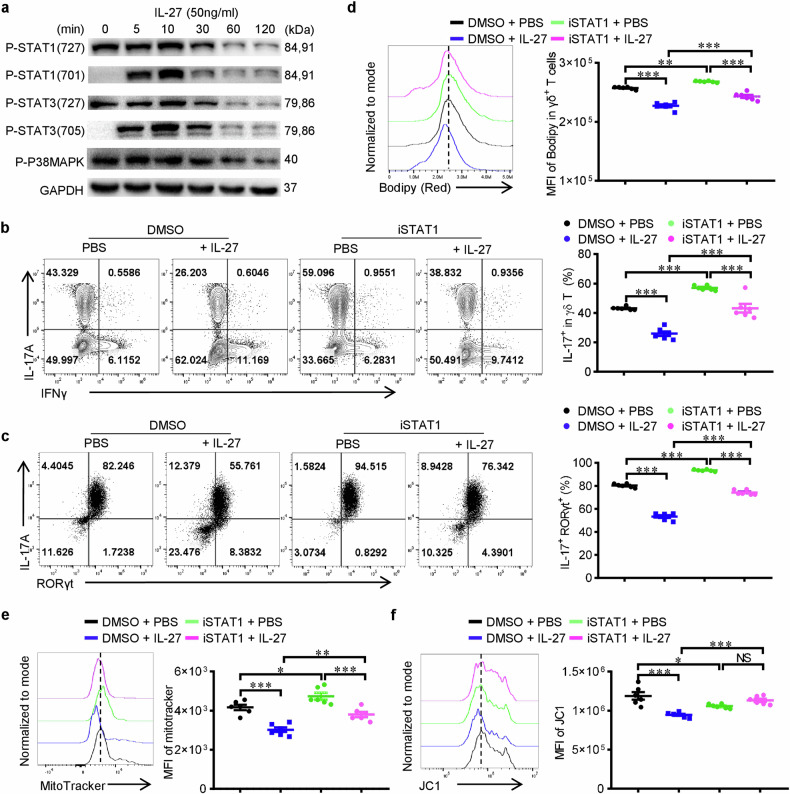
IL-27 restrains lipid anabolism and mitochondrial activity partially via STAT1. **a** Freshly isolated γδ T cells from the spleen of WT mice were treated with rmIL-27 (50 ng/ml) for 0, 5, 10, 30, 60, and 120 min and used for the immunoblotting analysis of phosphorylation of STAT1, STAT3 and p38-MAPK. **b**, **c** Flow cytometry of IL-17 levels in WT γδ T cells that were differentiated in vitro under γδ T17 priming conditions in the presence or absence of STAT1 inhibitor (Fludarabine, 10 μM) with or without rmIL-27 (50 ng/ml) (*n* = 6). **d** Flow cytometry analysis of cellular lipid contents in WT γδ T cells that were differentiated in vitro under γδ T17 priming conditions in the presence or absence of STAT1 inhibitor (Fludarabine, 10 μM) with or without rmIL-27 (50 ng/ml) (*n* = 6). **e** MitoTracker or (**f**) JC-1 staining of WT γδ T cells that were differentiated in vitro under γδ T17 priming conditions in the presence or absence of STAT1 inhibitor (Fludarabine, 10 μM) with or without rmIL-27 (50 ng/ml) (*n* = 6). Data were presented as mean ± SEM, statistical differences were performed using One-way ANOVA (b-f). *p < 0.05, **p < 0.01, ***p < 0.001, NS not significant.

The canonical pathways that mediate the transduction of IL-27/IL-27R signaling are STAT1, STAT3 and p38 MAPK [12, 13]. STAT1 is well recognized by its inhibition of IL-17 and promotion of IFN-γ [25], STAT3 supports the production of IL-17 in Th17 cells and γδ T cells [26], while p38 MAPK also is involved in the promotion of IFN-γ by IL-27 [13, 27]. To investigate whether IL-27 inhibits γδ T17 through these pathways, we first tested the activation of these molecules upon IL-27 treatment on freshly isolated WT splenic γδ T cells. We found that γδ T cells expressed all these three molecules and had basic phosphorylation of STAT1 (serine-727 site), STAT3 (serine-727 site) and p38 MAPK, but not STAT1 (tyrosine-701 site) or STAT3 (tyrosine-705 site). Upon stimulation by IL-27, the levels of pSTAT1 (Tyr701) and pSTAT3 (Tyr705) were dramatically increased in 5 min, while the activation of pSTAT1 (Ser727), pSTAT3 (Ser727) and p-p38 MAPK were not intensified (Fig. 4a). These results indicated that IL-27 induced the activation of pSTAT1 (Tyr701) and pSTAT3 (Tyr705), but not pSTAT1 (Ser 727), pSTAT3 (Ser727) or p-p38 MAPK signaling. We then added STAT1 specific inhibitor (fludarabine) or STAT3 specific inhibitor (C188-9) in the presence or absence of IL-27 during the differentiation of γδ T17. As expected, inhibiting STAT1 significantly upregulated the generation of γδ T17 while inhibiting STAT3 led to downregulation of IL-17 (Fig. 4b, c and Supplementary Fig. 4), which was in line with previous studies that STAT1 inhibited but STAT3 promoted the expression of IL-17. Besides, inhibiting STAT1 partially rescued the downregulation of IL-17 by IL-27 (Fig. 4b, c), suggesting that the activation of STAT1 partially contributed to the inhibition of γδ T17 and there might be STAT1/STAT3/p38 independent pathways that were responsible for the reduction of IL-17 by IL-27.

Next, we tested whether the disturbance of lipid metabolism and reduction of mitochondrial oxidative metabolism by IL-27 were due to the activation of STAT1. By staining lipids with C11-BODIPY (581/591), we found that STAT1 inhibition reversed the reduction of lipid contents by IL-27 (Fig. 4d), suggesting that IL-27 restrains lipid anabolism at least partially via STAT1 signaling pathway. Besides, STAT1 inhibition also partially rescued the decreased mitochondrial mass and completely rescued the reduction of mitochondrial membrane potential (Fig. 4e, f). Collectively, these results revealed that IL-27 restrains lipid anabolism and mitochondrial activity of γδ T17 cells partially via STAT1.

### IL-27 ameliorates γδ T17 cells mediated psoriatic skin inflammation

**Fig. 5:**
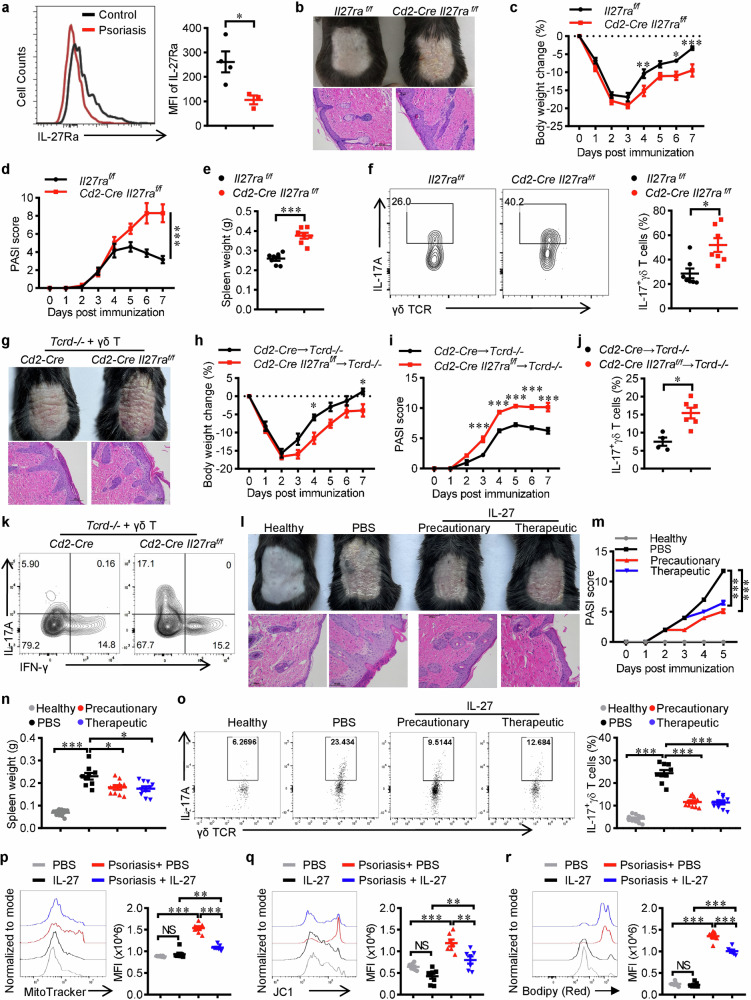
IL-27 ameliorates γδ T17 cells mediated psoriatic skin inflammation. **a** Flow cytometry of IL-27Ra levels in γδ T cells isolated from the skin tissues of healthy or IMQ-induced psoriasis mice. **b**–**f** *Cd2-Cre* and *Cd2-Cre Il27ra*^*flox/flox*^ mice (*n* = 7) were treated with IMQ daily for 7 days. **b** Representative images of dander and H&E-stained sections were shown. Scale bar, 100μm. **c** Body weight change. **d** PASI score. **e** Spleen weight. **f** Flow cytometry analysis of IL-17 in skin γδ T cells. **g**–**k** In vitro cultured γδ T cells from *Cd2-Cre* (*n* = 4) or *Cd2-Cre Il27ra*^*flox/flox*^ (*n* = 6) mice that were differentiated under γδ T17 priming condition were transferred into TCRδ-KO mice (5 × 10^5^ cells/mouse) two days before induction of psoriasis via IMQ. **g** Representative images of dander and H&E-stained sections were shown. Scale bar, 100 μm. **h** Body weight change. **i** PASI score. **j**, **k** Flow cytometry analyses of IL-17 in skin γδ T cells. **l**–**o** WT mice were intracutaneous administrated with rmIL-27 precautionarily (33.3 ng/kg/dose on Day −1, Day 1 and Day 3) or therapeutically (a single dose of injection on Day 3, 100 ng/kg) for the treatment of IMQ-induced psoriasis (*n* = 9 for PBS and *n* = 10 for the other groups). **l** Representative images of dander and H&E-stained sections were shown. Scale bar, 100 μm. **m** PASI score. **n** Spleen weight. **o** Flow cytometry analysis of IL-17 in skin γδ T cells. **p**–**r** Naïve or psoriatic mice were intracutaneous administrated with rmIL-27 on Day 3 post IMQ treatment (100 ng/kg), skin lymphocytes were isolated and dermal γδ T cells were analyzed (*n* = 7). Representative FACS plots and statistical analysis of mean fluorescent intensity for MitoTracker (**p**), JC-1 (**q**) and Bidipy (**r**). Data were presented as mean ± SEM, statistical differences were performed using two-tailed unpaired student’s *t*-test (**a**, **e**, **f**, **j**), One-way ANOVA (**n–r**) or Two-way ANOVA (**c**, **d**, **h**, **i**, **m**). *p < 0.05, **p < 0.01, ***p < 0.001, NS not significant.

As mentioned before, γδ T17 cells are kick-starters of proinflammatory responses such as skin psoriatic inflammation [5, 28]. *Tcrd-/-* mice developed much less severe psoriasis compared with WT controls [29, 30]. Thereby, we tested if IL-27 participates in the regulation of γδ T17 cells mediated inflammation. We first examined the expression of IL-27Ra in dermal γδ T cells in an IMQ-induced psoriasis mouse model, in which γδ T17 cells were significantly elevated and played a key role in driving skin inflammation [30, 31]. The expression of IL-27Ra was significantly decreased in γδ T cells from psoriatic skins compared with healthy controls (Fig. 5a), suggesting that the reduction of IL-27Ra might be involved in the regulation of γδ T17 cells mediated skin inflammation. Next, we induced psoriasis on *Cd2-Cre Il27ra*^*flox/flox*^ mice with *Il27ra*^*flox/flox*^ as controls. As expected, *Cd2-Cre Il27ra*^*flox/flox*^ mice showed aggravated pathogenesis as indicated by thicker acanthosis, more severe weight loss, higher PASI score, larger splenomegaly, and elevated γδ T17 percentage (Fig. 5b–f). Transferring in vitro differentiated γδ T17 cells that were isolated from the spleen of *Cd2-Cre Il27ra*^*flox/flox*^ mice into *Tcrd*^*−/−*^ mice also induced more severe psoriatic inflammation than γδ T cells from *Il27ra*^*flox/flox*^ controls (Fig. 5g–j). Moreover, precautionary or therapeutic intracutaneous injection of IL-27 proteins significantly ameliorated the pathogenesis of psoriasis with dramatic inhibition of γδ T17 cells (Fig. 5k–n) even though psoriatic γδ T cells had reduced expression of IL-27Ra (Fig. 5a). In line with the findings made in vitro, IL-27 treatment also disturbed lipid metabolism and restrained mitochondrial activity of dermal and splenic γδ T cells in mice with psoriasis (Fig. 5p–r and Supplementary Fig. 5). Collectively, these results indicated that IL-27 ameliorates γδ T17 cells mediated psoriatic skin inflammation.

## Discussion

γδ T cells provide effective immunosurveillance of xenobiotics and cellular damages in barrier tissues. γδ T17 cells are located in the dermis and lamina propria that beneath the epithelial layer and recruit neutrophils or adaptive immune cells to protect the host when encountering pathogens. However, excessive activation of γδ T17 cells by invaders, damaged cells or cytokines will kick-start proinflammatory responses such as skin psoriatic inflammation. Previous studies have demonstrated that γδ T17 cells strongly engaged oxidative metabolism, with increased mitochondrial mass and activity [8]. These cells overexpress the transcription factor NRF1 to promote the synthesis of proteins needed for mitochondrial biogenesis and OXPHOS [8], and inhibition of mitochondrial translation reduces γδ T17 and ameliorates imiquimod-induced psoriatic skin inflammation [10]. IL-27 has potent inhibitory effects on Th17-mediated immune responses, and Cao et al. have reported that IL-27 could suppress the development of γδ T17 cells in a postinfluenza pneumococcal pneumonia mouse model, which limited the protection against Streptococcus pneumoniae infection by γδ T17 cells [32]. However, the molecular machinery involved in the inhibition of γδ T17 by IL-27 are largely unknown. Besides, the role of IL-27 in γδ T17 cells mediated proinflammatory responses is undefined either. In this study, we found that IL-27 promotes lipolysis while inhibiting lipid synthesis, which leads to a reduced sum of lipids and the subsequent generation of phospholipids that are the main components of organelle membranes and cytomembranes. IL-27 also degenerates mitochondria and restrains oxidative metabolism, and the disturbance of lipid metabolism contributes to the dysfunction of mitochondria. Functionally, both disturbance of lipid metabolism and dysfunction of mitochondria contribute to the inhibition of γδ T17 by IL-27. More importantly, we found that the downregulation of IL-27Ra on γδ T cells is involved in the pathogenesis of psoriatic inflammation and IL-27 has preventive and therapeutic effects in ameliorating psoriasis.

The role of IL-27 in psoriasis is controversial. EL-Komy et al. [33] and Shibata et al. [34] reported significantly higher mean serum interleukin 27 levels in patients with psoriasis. EL-Komy et al. also found a negative correlation between serum IL-27 levels with disease severity [33]. While Chen et al. [35] and Michalak-Stoma et al. [36] reported downregulation of interleukin 27 in serum from psoriasis patients. Chen et al. also reported decreased IL-27 and IL-27Ra levels in the skin lesions of moderate-to-severe psoriasis patients [35]. Regarding the effects of IL-27 on the pathogenesis of psoriasis, Shibata et al. reported that IL-27 activated Th1-mediated responses to exacerbate IMQ-induced psoriasis-like skin lesions without affecting IL-17 [37], while Chen et al. found that subcutaneous administration of IL-27 recombinant protein lessened severity of IMQ-induced psoriasis-like cutaneous lesions by suppressing the production of IL-17 [35]. In our study, we found that the expression of IL-27Ra in dermal γδ T cells from mouse psoriatic skins was significantly decreased in comparison to healthy mice. *Il27ra* deficient splenic γδ T cells produced higher levels of IL-17 and were more pathogenic when transferred into *Tcrd-/-* mice. Intracutaneous injection of IL-27 inhibited the production of IL-17 from γδ T cells and ameliorated the pathogenesis of IMQ-induced psoriasis-like cutaneous lesions. These results strongly support a protective role of IL-27 in ameliorating psoriasis that is consistent with Chen’s work [35]. The reason for the discrepancy between our work and Shibata’s report [37] is currently unknown.

Recently, we found that IL-27 promotes the expenditure of lipids and energy dissipation as heat via uncoupling oxidative phosphorylation from ATP synthesis in adipocytes [14]. The mitochondrial proton leak effect during this process will also attenuate mitochondrial membrane potential [38]. Here, we also found that IL-27 promotes lipolysis, degenerates mitochondria and attenuates mitochondrial membrane potential in γδ T17 cells, suggesting similar activities of IL-27 on IL-27Ra-expressing cells. It’s worth checking whether IL-27 conducts its known actions on CD4 T cells and dendritic cells (DC) through these mechanisms.

In summary, these studies revealed that IL-27 promotes lipolysis, restrains phospholipid anabolism and reduces mitochondrial oxidative metabolism to inhibit γδ T17 mediated inflammation. This work uncovers the metabolic basis for the immune regulatory activity of IL-27 and provides novel insights into IL-27/IL-27Ra signaling, γδ T17 biology and the pathogenesis of psoriasis.

### Supplementary information

Supplementary Figure & Legends

Supplementary Table 1

Supplementary Table 2

Supplementary Table 3

Un-cropped images of WB

## Data Availability

RNA seq data were deposited at the NCBI database to be publicly available with accession numbers SRR27385675-SRR27385678 and SRR27385153-SRR27385157. Lipidomic data were deposited at MetaboLights database to be publicly available with accession numbers MTBLS9272. All other data are available from the corresponding authors upon request.
