# Supplementary material for: IL-27 disturbs lipid metabolism and restrains mitochondrial activity to inhibit γδ T17 cell-mediated skin inflammation
Source: Cell Death Dis. 2024 Jul 9;15(7):491. doi: 10.1038/s41419-024-06887-0 (PMC11233514; doi:10.1038/s41419-024-06887-0)
Supplement: Supplementary file 1 — Supplementary Figure & Legends [file 41419_2024_6887_MOESM1_ESM.pdf]

Supplementary Figure and Figure Legends

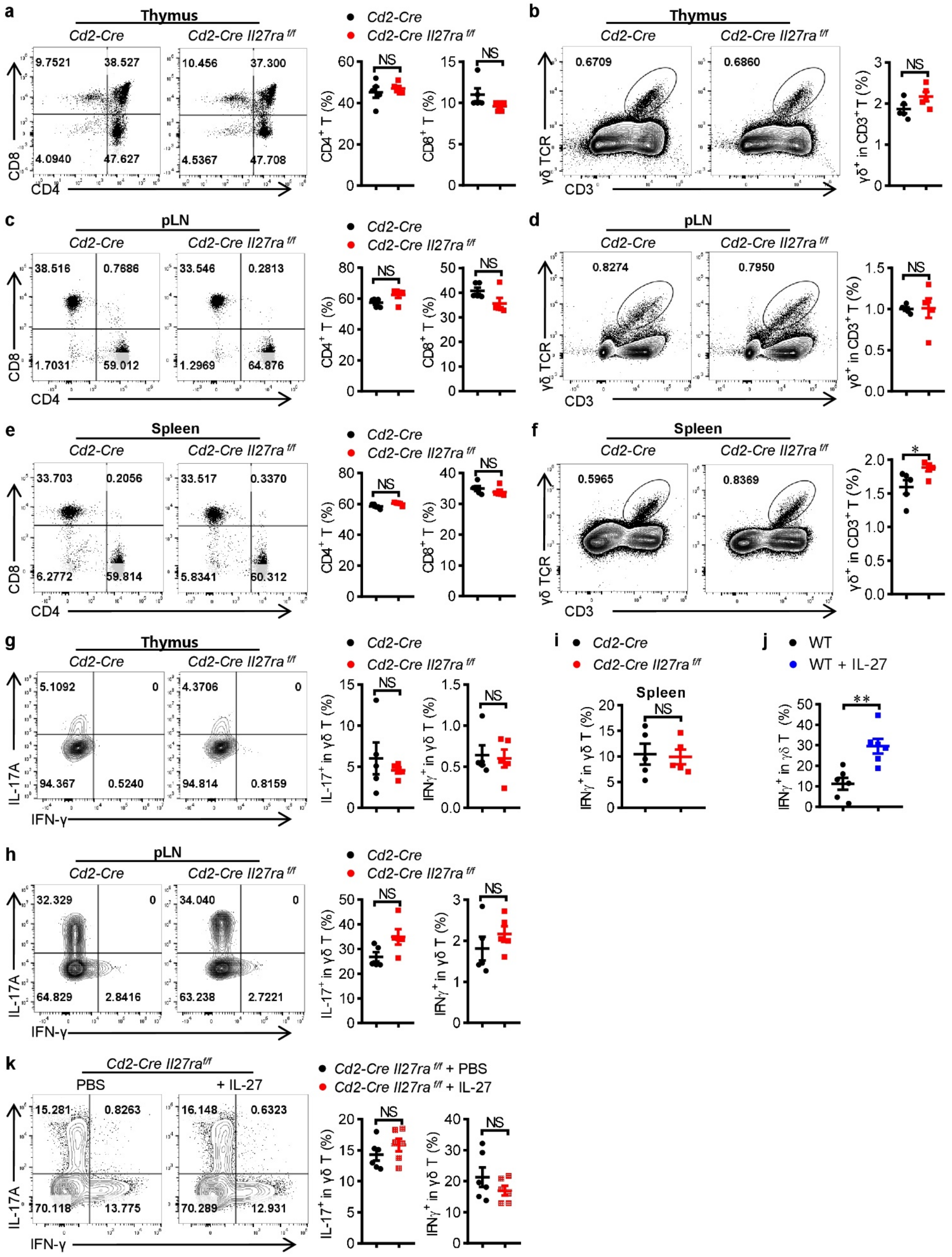

Figure S1. Characteristics of γδ T cells from *Cd2-Cre Il27ra$^{flox/flox}$* mice. (Refer to Figure 1)

(**a-b**) The percentages of $CD4^+$ and $CD8^+$ T cells (**a**) or γδ T cells (**b**) in the thymus of *Cd2-Cre Il27ra$^{flox/flox}$* or *Cd2-Cre* mice at 8-10 weeks of age (n = 5).

(**c-d**) The percentages of $CD4^+$ and $CD8^+$ T cells (**c**) or γδ T cells (**d**) in the peripheral lymph nodes of *Cd2-Cre Il27ra$^{flox/flox}$* or *Cd2-Cre* mice at 8-10 weeks of age (n = 5).

(**e-f**) The percentages of $CD4^+$ and $CD8^+$ T cells (**e**) or γδ T cells (**f**) in the spleen of *Cd2-Cre Il27ra$^{flox/flox}$* or *Cd2-Cre* mice at 8-10 weeks of age (n = 5).

(**g**) The percentages of IL-17^+ or IFN-γ^+ γδ T cells in the thymus of *Cd2-Cre Il27ra$^{flox/flox}$* or *Cd2-Cre* mice at 8-10 weeks of age (n = 5).

(**h**) The percentages of IL-17^+ or IFN-γ^+ γδ T cells in the peripheral lymph nodes of *Cd2-Cre Il27ra$^{flox/flox}$* or *Cd2-Cre* mice at 8-10 weeks of age (n = 5).

(**i**) Flow cytometry analysis of IFN-γ production by spleen γδ T cells from *Cd2-Cre* and *Cd2-Cre Il27ra$^{flox/flox}$* mice (n = 5).

(**j**) Splenocytes from WT mice were cultured under γδ T17 priming conditions with or without rmIL-27 (50ng/ml), and the production of IFN-γ from γδ T cells was detected and shown (n = 6).

(**k**) Splenocytes from *Cd2-Cre Il27ra$^{flox/flox}$* mice were cultured under γδ T17 priming conditions with or without rmIL-27 (50ng/ml) for 4 days, the production of IL-17A and IFN-γ from γδ T cells were detected and shown (n = 6).

Data were presented as mean ± SEM, statistical differences were performed using Two-tailed unpaired student's t-test (**a-k**). *$p < 0.05$, **$p<0.01$, NS, not significant.

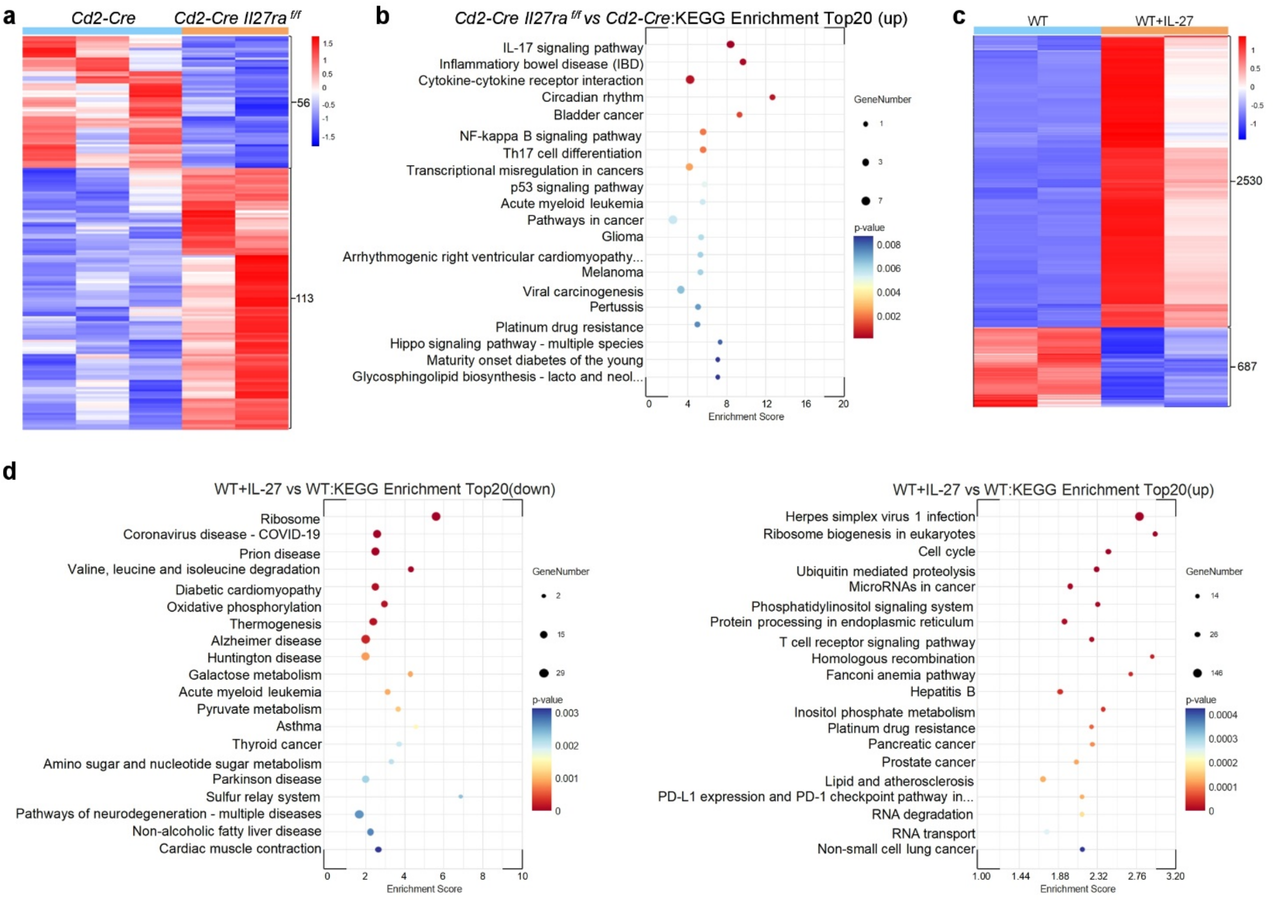

Figure S2. IL-27 affects the transcriptome of γδ T cells. (Refer to Figure 2)

(**a-b**) RNA-sequencing analysis was performed using *in vitro* differentiated γδ T17 cells from the spleen of *Cd2-Cre Il27ra$^{flox/flox}$* (n = 2) or *Cd2-Cre* (n = 3) mice. (**a**) Heatmap of differentially expressed genes. (**b**) Top 20 KEGG pathways that were significantly upregulated in IL-27Ra-deficient γδ T cells.

(**c-d**) RNA-sequencing analysis was performed using WT γδ T cells that were differentiated *in vitro* under γδ T17 condition with or without IL-27 (50ng/ml) treatment (n = 2). (**c**) Heatmap of differentially expressed genes. (**d**) Top 20 KEGG pathways that were significantly altered after IL-27 treatment.

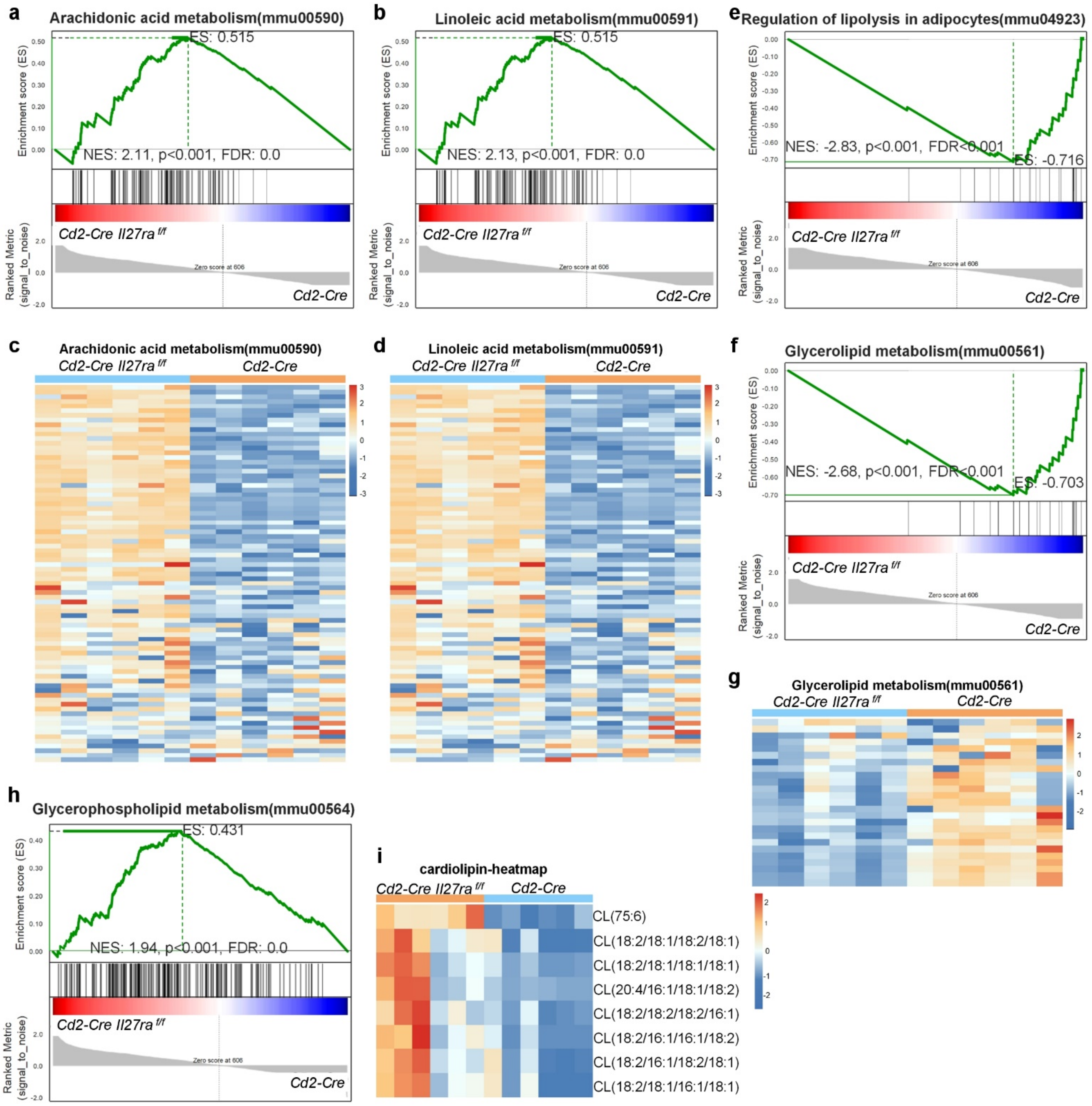

Figure S3. IL-27 disturbs lipid metabolism and restrains the generation of phospholipids in γδ T17 cells. (Refer to Figure 2)

Lipids were extracted from *in vitro* differentiated γδ T17 cells that were isolated from the spleen of *Cd2-Cre Il27ra*$^{flox/flox}$ or *Cd2-Cre* mice and used for lipidomic analysis via LC-MS (n=6). Metabolite set enrichment analysis of significantly altered lipids involved in Arachidonic acid metabolism (**a**), Linoleic acid metabolism (**b**), Lipolysis (**e**), Glycerolipid metabolism (**f**) and Glycerophospholipid metabolism (**h**). Heatmap of significantly altered lipids involved in Arachidonic acid metabolism (**c**), Linoleic acid metabolism (**d**), Glycerolipid metabolism (**g**) and Cardiolipin (**i**).

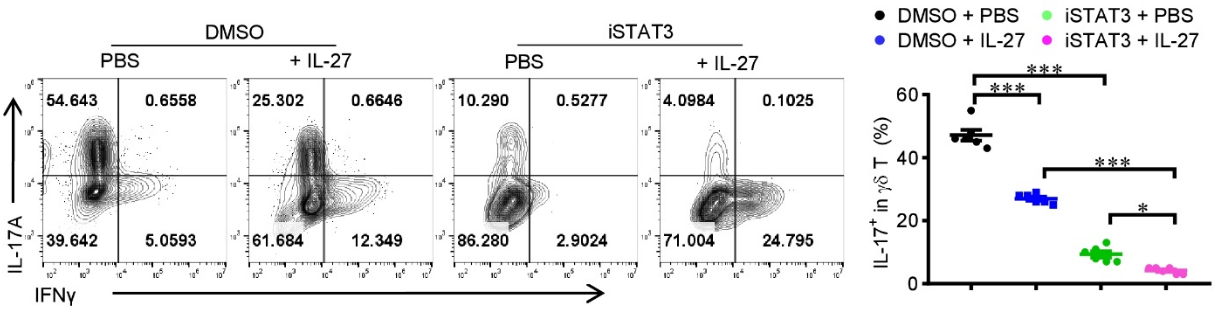

Figure S4. Inhibiting STAT3 reduces the differentiation of γδ T17 cells *in vitro*. (Refer to Figure 4)

Flow cytometry of IL-17 levels in WT γδ T cells that were differentiated *in vitro* under γδ T17 priming conditions in the presence or absence of STAT3 inhibitor (C188-9, 10 μM) with or without rmIL-27 (50ng/ml) (n = 6).

Data were presented as mean ± SEM, statistical differences were performed using One-way ANOVA. *p < 0.05, ***p < 0.001.

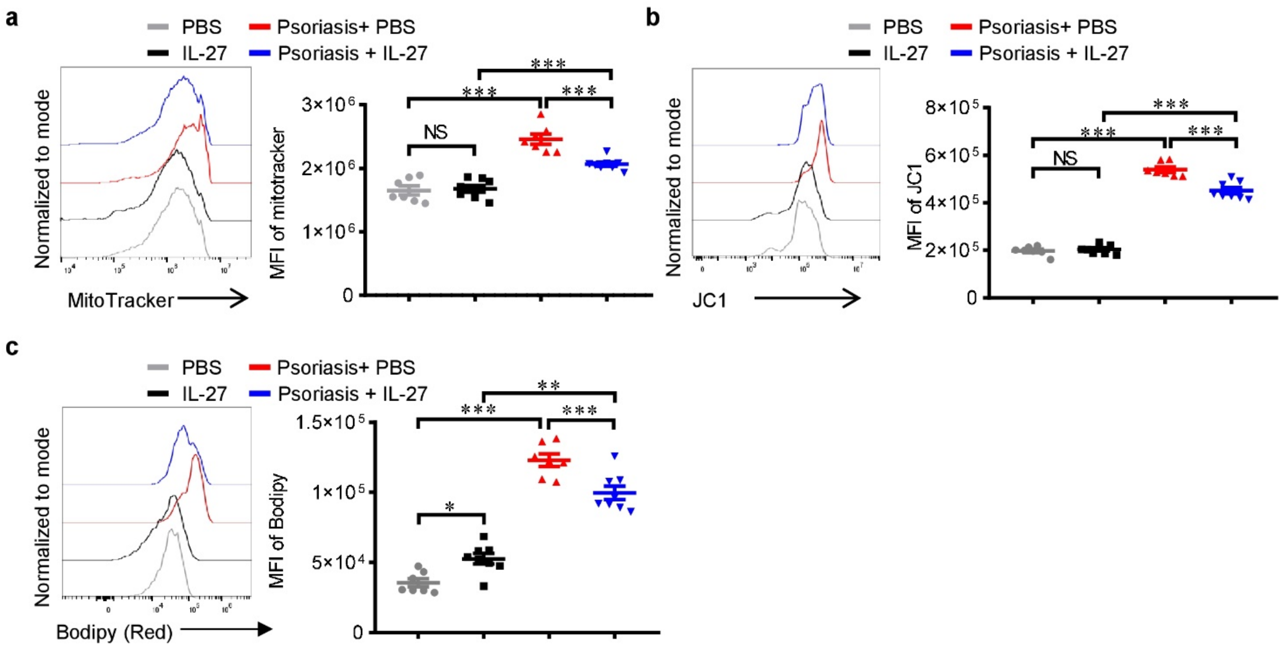

Figure S5. IL-27 disturbs lipid metabolism and restrains mitochondrial activity of psoriatic γδ T cells *in vivo*. (Refer to Figure 5)

Naïve or psoriatic mice were intracutaneous administrated with rmIL-27 on Day 3 post IMQ treatment (100ng/kg) as in **Figure 5**, splenocytes were isolated and γδ T cells were analyzed (n = 7 for PBS and n = 8 for IL-27). Representative FACS plots and statistical analysis of mean fluorescent intensity for MitoTracker (**a**), JC-1 (**b**) and Bidipy (**c**). Data were presented as mean ± SEM, statistical differences were performed using One-way ANOVA (**a-c**). *$p < 0.05$, **$p<0.01$, ***$p < 0.001$, NS, not significant.
