# Supplementary material for: IL-27 disturbs lipid metabolism and restrains mitochondrial activity to inhibit γδ T17 cell-mediated skin inflammation
Source: Cell Death Dis. 2024 Jul 9;15(7):491. doi: 10.1038/s41419-024-06887-0 (PMC11233514; doi:10.1038/s41419-024-06887-0)

Figure 2c. Un-cropped images of Western Blots

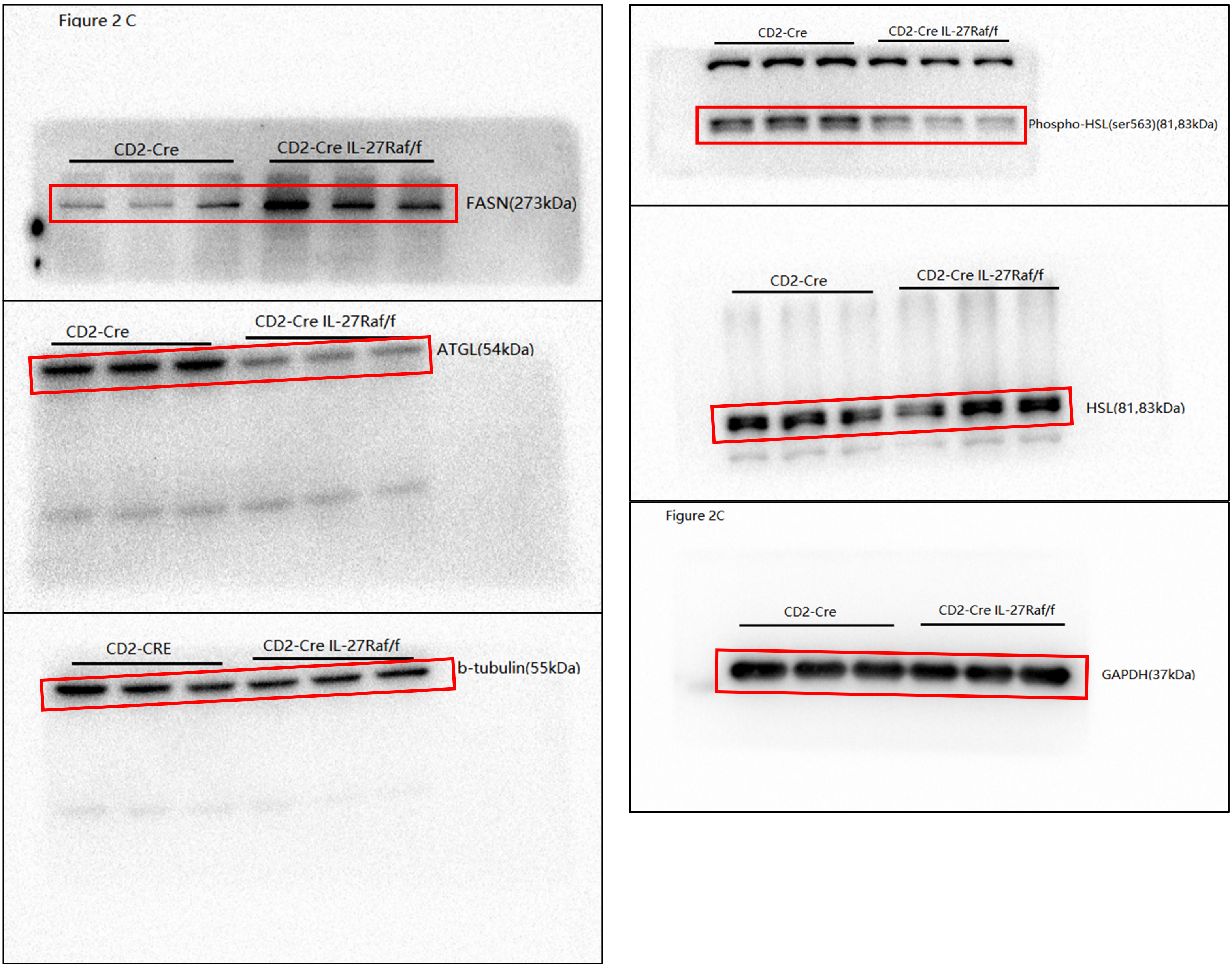

Figure 3f. Un-cropped images of Western Blots

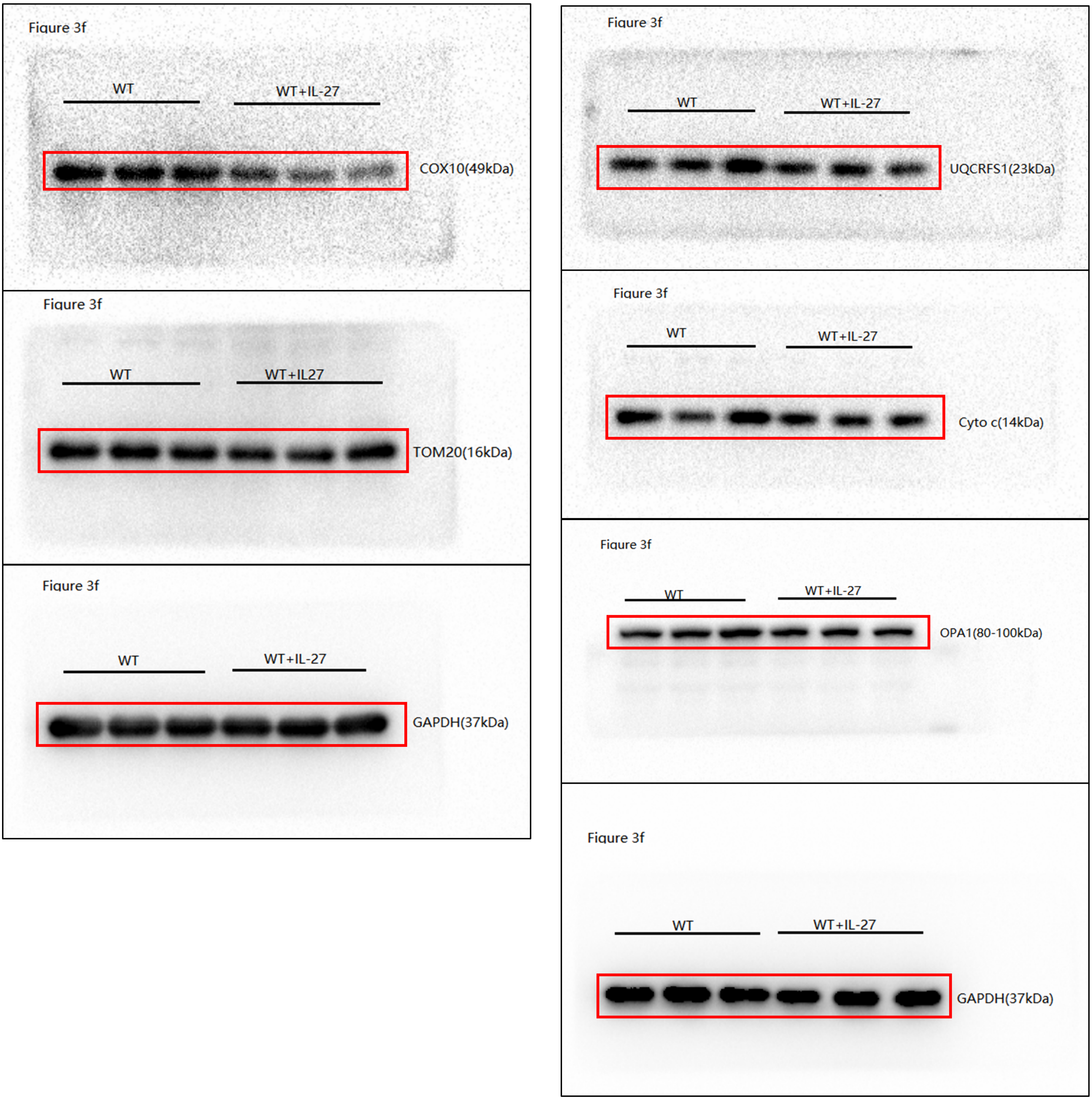

Figure 3f. Un-cropped images of Western Blots

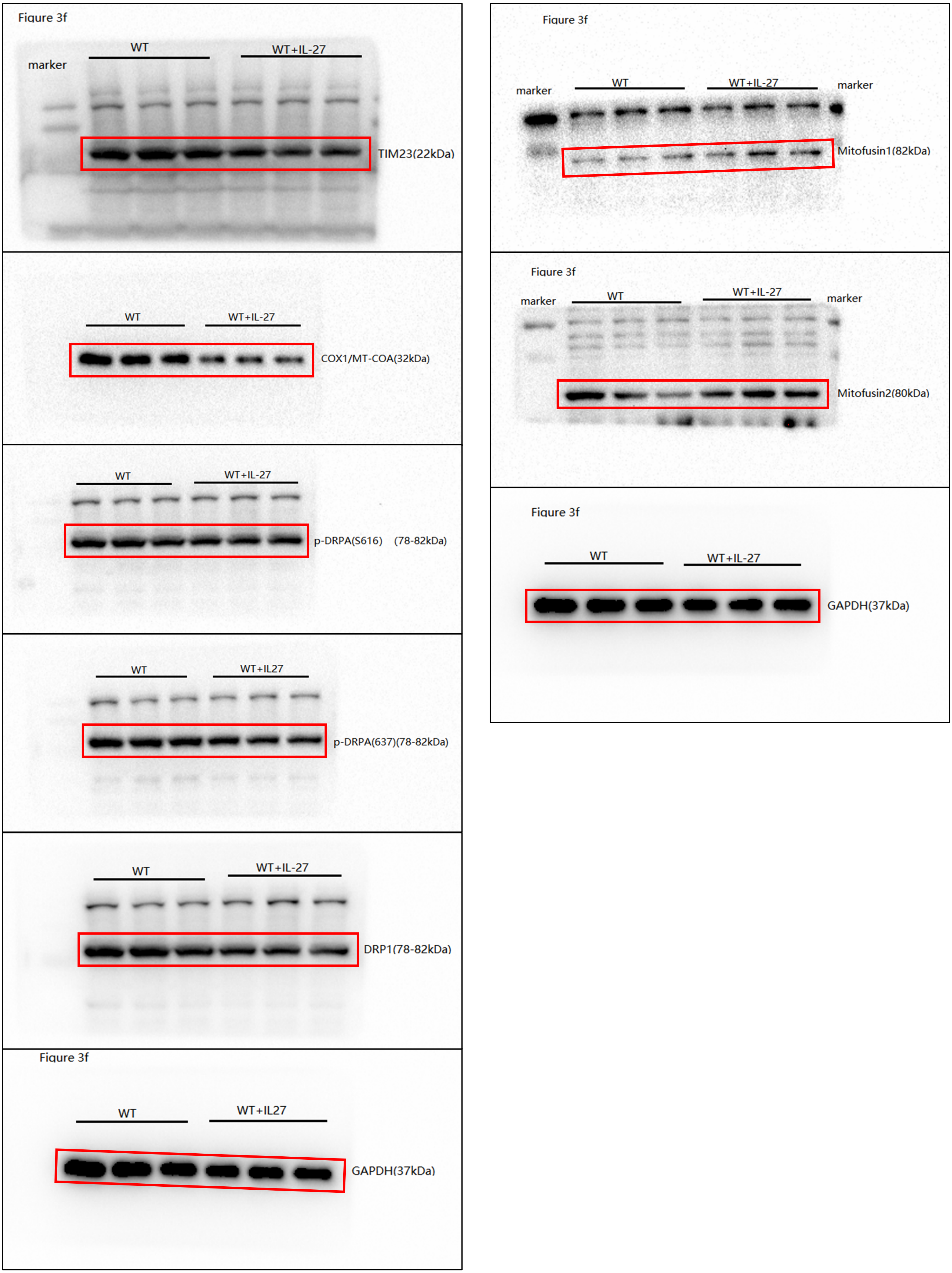

Figure 3f. Un-cropped images of Western Blots

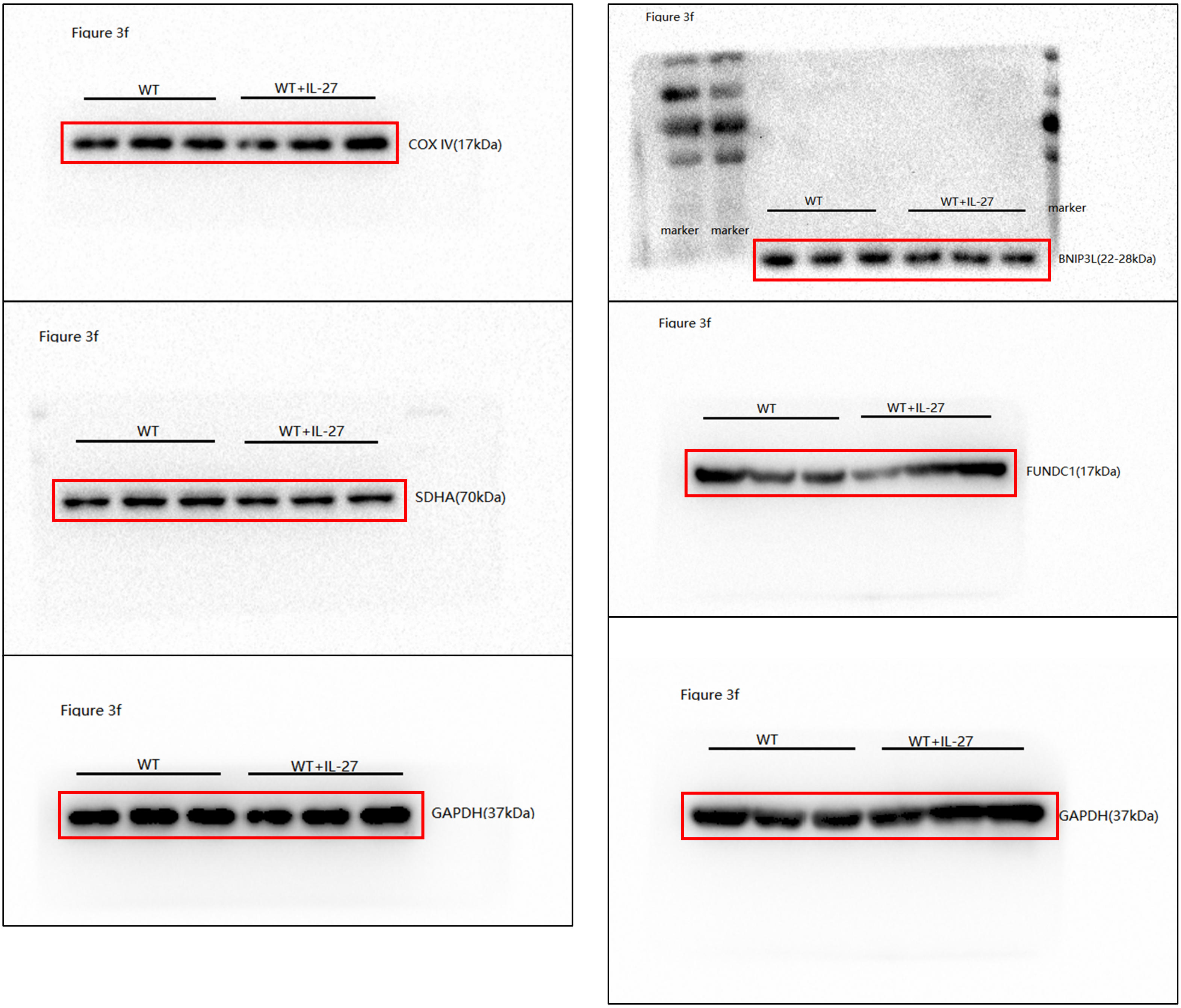

Figure 4a. Un-cropped images of Western Blots

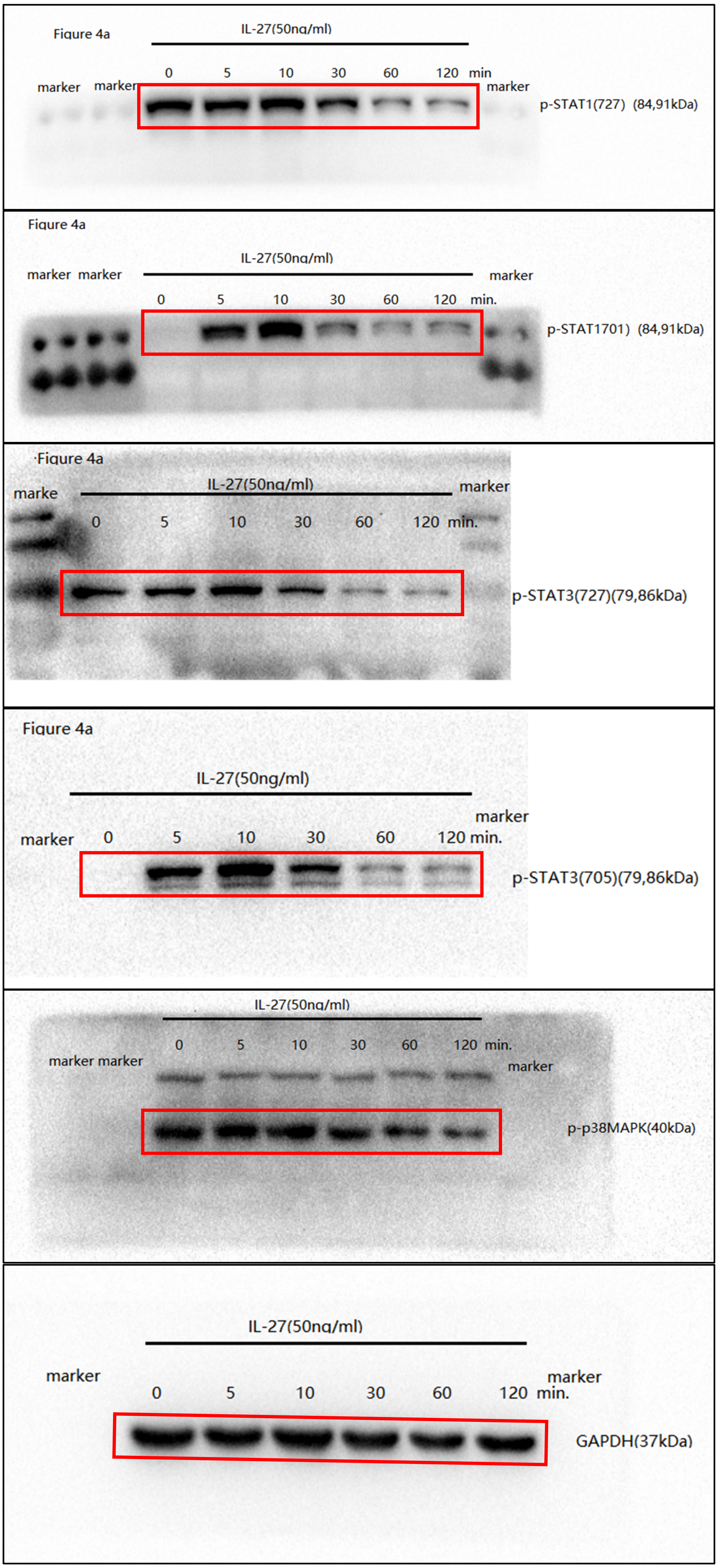

Supplement: Supplementary file 5 — Un-cropped images of WB [file 41419_2024_6887_MOESM5_ESM.pdf]
